# Exceptional phonon point versus free phonon coupling in Zn_1−x_Be_x_Te under pressure: an experimental and ab initio Raman study

**DOI:** 10.1038/s41598-022-04815-w

**Published:** 2022-01-14

**Authors:** M. B. Shoker, T. Alhaddad, O. Pagès, V. J. B. Torres, A. V. Postnikov, A. Polian, R. Hajj Hussein, G. K. Pradhan, C. Narayana, C. Gardiennet, G. Kervern, L. Nataf, S. Ravy, J.-P. Itié, K. Strzałkowski, A. Marasek, F. Firszt

**Affiliations:** 1 Université de Lorraine, LCP-A2MC, ER 4632, 57000 Metz, France; 2 Departamento de Fisica and I3N, Universidade de Aveiro, 3810-193 Aveiro, Portugal; 3 Institut de Minéralogie, de Physique des Matériaux et de Cosmochimie, Sorbonne Université — UMR CNRS 7590, 75005 Paris, France; 4 Synchrotron SOLEIL, L’Orme des Merisiers Saint-Aubin, BP 48, 91192 Gif-sur-Yvette Cedex, France; 5 Department of Physics, School of Applied Sciences, KIIT Deemed to be University, Bhubaneswar, Odisha 751024 India; 6 Jawaharlal Nehru Centre for Advanced Scientific Research (JNCASR), Jakkur P.O., Bangalore, 560064 India; 7 Laboratoire de Cristallographie, Résonance Magnétique et Modélisations, UMR 7036, Université de Lorraine, 54506 Vandoeuvre-lès-Nancy, France; 8 Faculty of Physics, Astronomy and Informatics, Institute of Physics, Nicolaus Copernicus University in Toruń, ul. Grudziądzka 5, 87-100 Toruń, Poland

**Keywords:** Materials science, Physics

## Abstract

Raman scattering and ab initio Raman/phonon calculations, supported by X-ray diffraction, are combined to study the vibrational properties of Zn_1−x_Be_x_Te under pressure. The dependence of the Be–Te (distinct) and Zn–Te (compact) Raman doublets that distinguish between Be- and Zn-like environments is examined within the percolation model with special attention to x ~ (0,1). The Be-like environment hardens faster than the Zn-like one under pressure, resulting in the two sub-modes per doublet getting closer and mechanically coupled. When a bond is so dominant that it forms a matrix-like continuum, its two submodes freely couple on crossing at the resonance, with an effective transfer of oscillator strength. Post resonance the two submodes stabilize into an inverted doublet shifted in block under pressure. When a bond achieves lower content and merely self-connects via (finite/infinite) treelike chains, the coupling is undermined by overdamping of the in-chain stretching until a «phonon exceptional point» is reached at the resonance. Only the out-of-chain vibrations «survive» the resonance, the in-chain ones are «killed». This picture is not bond-related, and hence presumably generic to mixed crystals of the closing-type under pressure (dominant over the opening-type), indicating a key role of the mesostructure in the pressure dependence of phonons in mixed crystals.

## Introduction

In the race between ZnSe-based II–VI and GaN-based III–V semiconductors to achieve blue lasers robust in time, one option to remedy the naturally soft/ionic II–VI lattice via alloying with stiff/covalent Be-based bonds lead to the emergence of Zn_1−x_Be_x_-chalcogenides in the late nineties^1,2^. These systems are also interesting on the fundamental side. In particular, the large difference in bond physical properties^3^ (length, stiffness, effective mass) exacerbates the phonon properties, offering a chance to achieve an understanding of the phonon mode behavior of mixed crystals beyond the traditional classification (including four main types^4–6^: one-mode, two-mode, intermediary one/two-mode and multi-mode). First, the stiff/short/light Be-VI and soft/long/heavy Zn-VI bonds vibrate at far-off frequencies—separated by as much as $\sim$ 200 cm^−1^. This is ideal to assess how each bond vibration is specifically impacted by alloying. Moreover, Zn_1−x_Be_x_-chalcogenides crystallize in a structure (zincblende) with maximal (cubic) symmetry. This results in an ultimately simple vibration pattern, which helps to achieve a clear understanding.

Remarkably, Zn_1−x_Be_x_Se and Zn_1−x_Be_x_Te exhibit a distinct bimodal pattern per bond in their Raman spectra^7^, falling out of the scope of the historical Modified Random Element Isodisplacement (MREI)^8^ and cluster^9^ models supporting the above classification. This was explained by introducing our so-called percolation model in the past few years. Subsequently, the percolation model applied to all tested IV, III–V and II–VI mixed crystals^10^, suggesting its universal character and, hence, the possible vacuity of the above classification.

Generally, the percolation model assumes the sensitivity of a bond vibration to its local AC- or BC-like environment in zincblende A_1−x_B_x_C mixed crystals (1-bond $\to$ 2-mode behavior), as defined up to first/second-neighbors at most. This is consistent with a basic feature of the bond charge model used to describe the phonon dispersion of the IV^11^, III–V^12^ and II–VI^13^ semiconductor compounds whose phonons are essentially a matter of short range interactions. Besides, each percolation-type oscillator, representing a bond in a given environment, is defined at one dimension (1D) along with the equations of motion per atom. The scalar approach is justified because Raman scattering operates near the centre $\Gamma$ (q = 0) of the Brillouin zone (due to the quasi vertical dispersion of the laser probe) where the atom displacements duplicate from one lattice unit cell to another. This suppresses the need to spot an atom in the real three-dimensional (3D) crystal. Last, the Raman doublet is best resolved for the short/stiff/light bond that vibrates at high frequency because the light substituent (say A) has a small covalent radius. As such, it has more freedom than the large/heavy substituent (B) to move around in its C-cage, as needed to accomodate the local contrast in the (A-C, B-C) bond physical properties, with concomitant impact on the A-C Raman frequencies, being more diversified. In fact, the long/soft/heavy bond (B-C) remains generally blind to its local environment (1-bond $\to$ 1-mode behavior). This results in an apparent three-mode [1 $\times$(B-C), 2 $\times$(A-C)] Raman behavior in total for a A_1−x_B_x_C zincblende mixed crystal. The only known exception of a canonical four-mode Raman pattern is Zn_1−x_Be_x_Te—studied in this work—in which a compact Zn–Te doublet is resolved experimentally beside the distinct Be–Te one^7^.

Besides its fundamental interest, the percolation model opens up a number of applications, outlined below.

First, equipped with a relevant order parameter ($\kappa$) it can be used to shed light upon the nature of the A $\leftrightarrow$ B substitution in ternary-zincblende A_1−x_B_x_C and binary-diamond A_1−x_B_x_ mixed crystals—as to whether this is random ($\kappa$ = 0) or not, i.e., due to clustering ($\kappa$ > 0) or anticlustering ($\kappa$ < 0). A direct insight is achieved by comparing the Raman intensities of the two sub-modes forming a given Raman doublet^14^.

Second, the distinct Raman doublet due to the short bond offers a sensitive chemical probe to study the pressure dependence of the vibrational properties of the zincblende-type mixed crystals at the local scale. A recent test on the way towards the zincblende $\to$ rock-salt transition of various ZnSe-based mixed crystals (including Zn_1−x_Be_x_Se)^10^ revealed that the Raman doublet of the native zincblende phase is reactive to pressure. It closes or opens depending on the difference between the pressure-induced hardening rates of the AC- and BC-like environments behind the doublet, as governed by the volume derivative of the ionicity ($\frac{\partial {f}_{i}}{\partial lnV}$, using the same notation as in Ref.^3^) of the corresponding host-bond species^3^. This we refer to as the $\frac{\partial {f}_{i}}{\partial lnV}$-mechanism below.

The latter mechanism suffices to explain all of the attributes of the pressure-induced closing/opening process observed in the zincblende phase^10^ of the studied mixed crystals so far, irrespectively of any consideration relative to the symmetry of the final (pressure-induced) crystal phase (rock-salt in this case)^10^. Hence, this process does not inform on the pressure-induced structural transition per se. This merely acts as a limiting factor likely to interrupt the (zincblende) process.

Based on the given $\frac{\partial {f}_{i}}{\partial lnV}$-mechanism for closing/opening, a partition of II–VI and III–V zincblende-type mixed crystals has been derived recently^10^. Remarkably, in all studied mixed crystals so far, the closing ended up at the crossing into a so-called «exceptional phonon point». This reflects an exact compensation of the mechanical coupling (gain) that develops between the two like sub-oscillators forming a percolation-type Raman doublet when forced into proximity by pressure, due to overdamping (loss) of one sub-oscillator until its full extinction/freezing at the resonance^10^.

**Figure 1:**
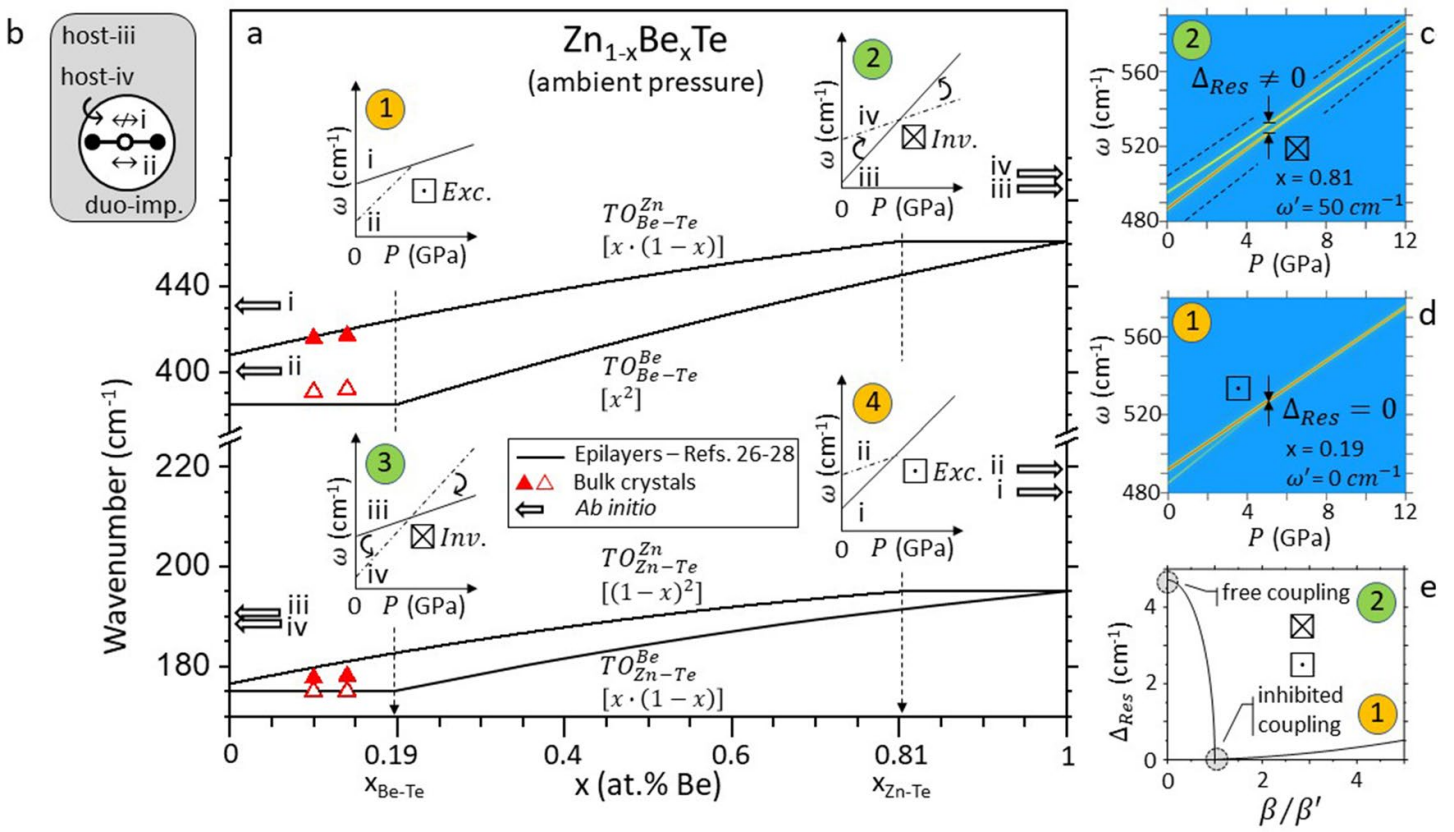
Zn_1−x_Be_x_Te Percolation scheme at ambient pressure. **(a)** Composition dependence of the Zn_1−x_Be_x_Te TO branches (plain lines) schematically derived from Raman frequencies obtained with epitaxial layers (available in Refs.^26–28^). The current data obtained with bulk crystals are superimposed (triangles), for comparison. A distinction is done between vibrations in Zn- (upper branches, full symbols) and Cd-like (lower branches, hollow symbols) environments, defined at the first-neighbor scale. The related fractions of 1D-oscillators, impacting the TO Raman intensities, are specified in square brackets. The pressure-induced closing processes of various doublets depending on bond content are schematically indicated (①-to-④), emphasizing the free (⊠) or inhibited (⊡) coupling at the resonance (Res.), resulting in inversion ($Inv.$) of the doublet, or extinction (*Ext.*) of one submode, respectively. In each panel the dominant/minor modes are distinguised using full/dashed-dotted lines. In the free-coupling regimes ➁ and ➂ an effective transfert of oscillator strength is indicated by curved arrows, further specifying the sense of transfert. In the inhibited-coupling regimes ① and ④, the absence of arrows reflects an absence of transfert. **(b)** Ab initio (AIMPRO) insight (large arrows) into the four asymptotic $\left\{{TO}_{Zn-Te}^{Be},{TO}_{Zn-Te}^{Zn},{TO}_{Be{{-}}Te}^{Be},{TO}_{Zn-Te}^{Zn}\right\}$ frequencies due to parent-like supercells containing a duo of impurities (x ~ 0,1), referring to an impurity bond (i,ii) or a host bond (iii,iv) vibrating in their like (ii,iii) or foreign (i,iv) environment, as sketched out. **(c,d)** Raman cross sections reflecting the pressure-induced closure of the Be-Te doublet in the inhibited ① and free ➁ coupling regimes, respectively. In the latter case, dotted lines help visualize (qualitatively) the cone-shape frequency domain in which the coupling takes place (see text). In both panels red/yellow glows reflect large/small Raman intensities. **(e)** The two regimes refer to extreme cases in the overdamping vs. coupling competition (captured via ${\beta }^{{\prime}}/\beta$), impacting the frequency gap between the normal modes of the coupled system at the resonance (${\Delta }_{Res}$), as indicated (shaded areas).

Though Ref.^10^ introduced a bipartition of mixed crystals, it still reflects a limited understanding solely concerned with the phonon mode behavior of the short bond species when this is minor in the crystal (corresponding to regime ① in Fig. 1). In this work we further explore the second application with the aim to achieve a full/synthetic picture (further covering the remaining regimes ➁, ➂ and ④ in Fig. 1) for the pressure-induced closure process encompassing both bonds of a mixed crystal, taken as the minor or dominant species.

For doing so we address more specifically two pending issues behind Ref.^10^, listed hereafter by focusing on the leading Zn_1−x_Be_x_Se system. Due to the limited Be incorporation in ZnSe with the used growth (Bridgman) method^15^, the closing of the (distinct) Raman doublet due to the short Be–Se bond could be studied only when the Be-Se bonds self-connect into (finite or infinite) treelike chains, i.e., their basic arrangement when the Zn–Se bonds percolate (x $\le$ x_Zn−Te_ = 0.81, referring to the Zn–Te bond percolation threshold)^16^. It does not make physical sense that the Be-Se freezing persists when the Be–Se bonds become so dominant as to form a matrix-like continuum for a dispersion of ZnSe-treelike chains (x $\ge$ 0.81). One may well wonder what scenario substitutes for the phonon freezing then. Second, according to the given rule above, the (compact) Raman doublet due to the (long) Zn–Se bond should also close under pressure, as the (distinct) Be-Se one. However, this contradicts experimental findings (see Fig. S1 of Supplementary Sect. I). Now, these may not be fully reliable because the one-phonon $\Gamma$-like Zn–Se Raman signal is well-known to interfere with various zone-edge two-phonon transverse (2TA) and longitudinal (TA + LA) acoustic continua in Zn_1−x_Be_x_Se^17^.

The above two pending issues are tackled below by shifting the focus from ZnSe-based systems to the leading percolation-type ZnTe-based one, i.e., Zn_1−x_Be_x_Te. Both its Be- and Zn-based Raman doublets are expected to close under pressure^10^, just like with Zn_1−x_Be_x_Se, so that a critical comparison is possible. Moreover, the zone-edge two-phonon acoustic bands are detuned from the zone-center Raman signal in ZnTe^13,18^. Hence we have good hope to achieve a reliable experimental insight into the pressure dependence of the (compact) Raman doublet due to the long Zn-based bond.

We perform a high-pressure Raman study of high-quality free-standing Zn_1−x_Be_x_Te bulk crystals with moderate Be content under the current limit of ~ 21 at% achievable with the used growth (Bridgman) method. Despite the moderate Be content, both the Zn–Te and Be–Te Raman doublets are visible and can be studied up to the first pressure-induced structural transition, independently identified around 10 GPa (as with ZnTe^19,20^) by X-ray diffraction. The lacking experimental insight at large Be content is searched for by performing ab initio calculations of the high pressure Raman spectra (Ab initio Modeling PROgram—AIMPRO^21,22^ code) and of the equivalent (in a crude approximation) high-pressure $\Gamma$-projected phonon density of states ($\Gamma$-PhDOS, SIESTA^23^ code) to large (216- and 64-atom, correspondingly) supercells hosting the prototypical percolation-type impurity motif, i.e., a duo of impurities sitting next to each other. The experimental and ab initio trends are jointly discussed within a simple model of coupled-damped harmonic 1D-oscillators—for the sake of consistency with the 1D-percolation scheme—in the spirit of a recent approach developed by Dolfo and Vigué^24^.

## Results and discussion

The relevant pressure domain for the high-pressure Zn_1−x_Be_x_Te (x = 0.11, 0.14) Raman study is determined by high-pressure X-ray diffraction at the CRISTAL beamline of synchrotron SOLEIL using a Zn_0.86_Be_0.14_Te powder (see Fig. S3 of Supplementary Sect. II). The zincblende (Zb) structure is preserved until the mixed crystal transiently adopts the cinnabar (Cb) structure (~ 12.5 GPa) and stabilizes into the Cmcm one (~ 14.5 GPa), in line with the pressure-induced structural path taken by ZnTe (~ 9.5 GPa: Zb $\to$ Cb; ~ 10.5 GPa: Cb $\to$ Cmcm)^19,20,25^. The bulk modulus at ambient pressure B_0_ derived in the native zincblende phase at x = 0.14 from the lattice constant pressure dependence (see Fig. S4 of Supplementary Sect. II) complies with the linear dependence between the parent values, within error bars. This offers an insight into the Zn_0.86_Be_0.14_Te mechanical properties at the macroscopic scale, completing that gained hereafter at the bond scale by high-pressure Raman scattering.

A schematic overview of the 1-bond $\to$ 2-mode percolation-type Raman pattern of Zn_1−x_Be_x_Te at ambient pressure (plain lines), based on the transverse optic (TO) modes earlier detected with epitaxial layers^26–28^, is displayed in Fig. 1a, for reference pupose. Each TO is labeled with a subscript and a superscript specifying the bond vibration and its local environment, respectively.

The four-modes $\left\{{TO}_{Zn-Te}^{Be},{TO}_{Zn-Te,}^{Zn}{TO}_{Be{{-}}Te}^{Be},{TO}_{Be{{-}}Te}^{Zn}\right\}$ Raman signal detected at ambient pressure in the pure-TO backscattering geometry at normal incidence/detection onto/from (110) cleaved/edge faces with our Zn_0.89_Be_0.11_Te and Zn_0.86_Be_0.14_Te bulk crystals match quasi exactly those of epitaxial layers with similar composition (within 1%, see Fig. S6 of Supplementary Sect. II). In both doublets of epitaxial layers the Raman intensity ratio was shown to reflect a random Zn $\leftrightarrow$ Be substitution—assuming a sensitivity of bond vibrations up to first-neighbors^7^. We deduce the same for our bulk crystals, by analogy. The related fractions of 1D-oscillators behind the TO’s are indicated (in square brackets, Fig. 1a). An independent insight into the nature of the Zn $\leftrightarrow$ Be substitution searched for by ^125^Te solid-state nuclear magnetic resonance (shown in Fig. S5 of Supplementary Sect. II, for the sake of completeness) is difficult to decypher, presumably due to the dramatic contrast between the (Zn–Te, Be–Te) bond physical properties, resulting in a prohibitive signal distortion.

Ab initio support to the Zn_1−x_Be_x_Te TO-percolation scheme in the parent/impurity limits (x ~ 0 and 1)—where our experimental study takes place—is gained via ab initio (AIMPRO) calculations of the TO Raman spectra done on large (216-atom) parent-like supercells with zincblende structure containing the prototypical percolation-type impurity motif, i.e., a duo of connected (via Te) impurities (Fig. 1b). The out-of-chain (i, $\nleftrightarrow$) and in-chain (ii, $\leftrightarrow$) duo-modes together with the host-bond vibrations away from the duo (iii) and close to it (iv) address all asymptotic frequencies (large arrows, Fig. 1a) at x ~ (0,1) of the four $\left\{{TO}_{Zn-Te}^{Be},{TO}_{Zn-Te}^{Zn},{TO}_{Be{{-}}Te}^{Be},{TO}_{Zn-Te}^{Zn}\right\}$ branches. More detail is given at a later stage. Now, we only note that the ab initio frequencies at ambient pressure are consistent with experiment—if we omit a slight overestimate due to a well-known bias of the used linear density approximation (see “Methods” section) to enhance the bond force constants.

We cannot escape a brief discussion of the inverse (i)-to-(iv) scaling at both ends of the composition domain. A basic trend is used that the bond force constant reduces (enlarges) when a bond is stretched (compressed), with concomitant impact on the TO frequency, being decreased (increased). Consider, e.g., x ~ 0. An isolated Be–Te bond (short), subject to type-(i) vibrations only (in foreign environment), suffers a tensile strain from the ZnTe host matrix—hence (i) is below the parent ${TO}_{BeTe}$ mode (x ~ 1). A Be-duo has a lower symmetry than an isolated-Be impurity, so that it is less efficient in accommodating the tensile strain. Hence, the in-chain Be-Te bonds are longer than the isolated ones—verified by ab initio calculations^7,29^, resulting in (ii) below (i). Similarly, the long Zn–Te bonds close to the short Be–Te ones experience a tensile strain, and hence are softened with respect to the matrix-like Zn–Te bonds, resulting in (iv) below (iii). The same discussion can be transposed at x ~ 1. Only, the local strain is compressive in this case, leading to an inverse (i)-to-(iv) scaling.

**Figure 2:**
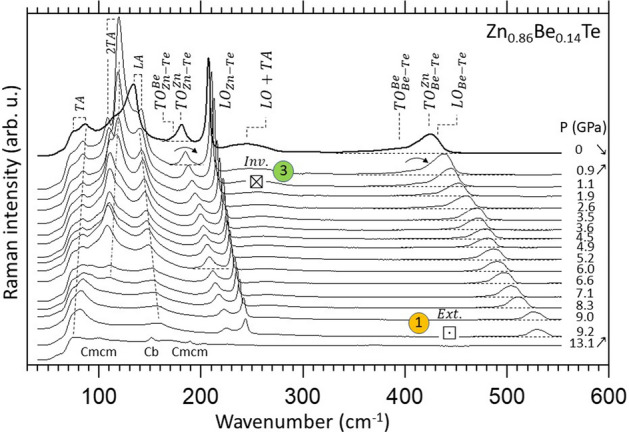
Selection of high-pressure Zn_0.86_Be_0.14_Te Raman spectra. The upstroke regime is emphasized (up-pointing arrow). The spectrum taken with pressure fully released (down-pointing arrow) is shown (top/thick curve), for reference purpose. The pressure-induced inversion (➂, ⊠, $Inv.$) and extinction (①, ⊡, $Ext.$) processes of the Zn–Te and Be–Te doublets are emphasized by curved arrows indicating the upward shift of the minor mode across (Zn–Te range) and up to (Be–Te range) the dominant one, using the same notation as in Fig. 1. Flat baselines are materialized (dashed lines) for a better resolution by eye of the Zn–Te and Be–Te TO doublets.

A selection of high-pressure Raman spectra taken with a non-oriented Zn_0.86_Be_0.14_Te crystal, offering simultaneous access to the longitudinal (LO) and transverse (TO) optic modes, is shown in Fig. 2 (similar Zn_0.89_Be_0.11_Te data are provided in Fig. S8 of Supplementary Sect. II). The Raman signal is stable in shape up to 9.2 GPa, and changes at 13.1 GPa. This is due to the structural transitions from the native zincblende phase to Cmcm via the transient cinnabar phase detected by X-ray diffraction. The main Raman peaks are assigned *per* phase as proposed by Camacho et al. in their high pressure Raman study of ZnTe^19,20^. We are mostly interested in the pressure dependence of the zincblende signal. As already mentioned the Zn–Te signal is not contaminated by the lower two-phonon acoustic bands at ambient pressure. The situation improves further under pressure since the acoustic bands soften (2TA, emphasizing the natural TA trend) or remain pinned at a (nearly) fixed frequency (TA-LA, due to neutralization of the LA-hardening by the TA-softening) whereas the optic modes harden^30,31^. As for the Be–Te signal, it emerges in a spectral range characterized by a nearly flat baseline. Altogether, this is ideal to achieve reliable insights into the Zn–Te and Be–Te signals depending on pressure.

Most semiconductor compounds become less ionic (more covalent) when compressed—the volume derivative of the bond ionicity $\frac{\partial {f}_{i}}{\partial lnV}$ is usually positive, resulting in enhanced mechanical properties^3^. In Zn_1−x_Be_x_Te, $\frac{\partial {f}_{i}}{\partial lnV}$ is larger for Be–Te (0.743) than for Zn–Te (0.553)^3^, meaning that the Be-like environment of a bond hardens faster than the Zn-like one under pressure. Hence both the Be-Te and Zn-Te Raman doublets should close under pressure, at any composition^10^. The pressure-induced Be–Te and Zn–Te closings are actually evidenced experimentally at moderate Be content (x = 0.14—Fig. 2, x = 0.11—Fig. S8), with important differences though, as anticipated.

**Figure 3:**
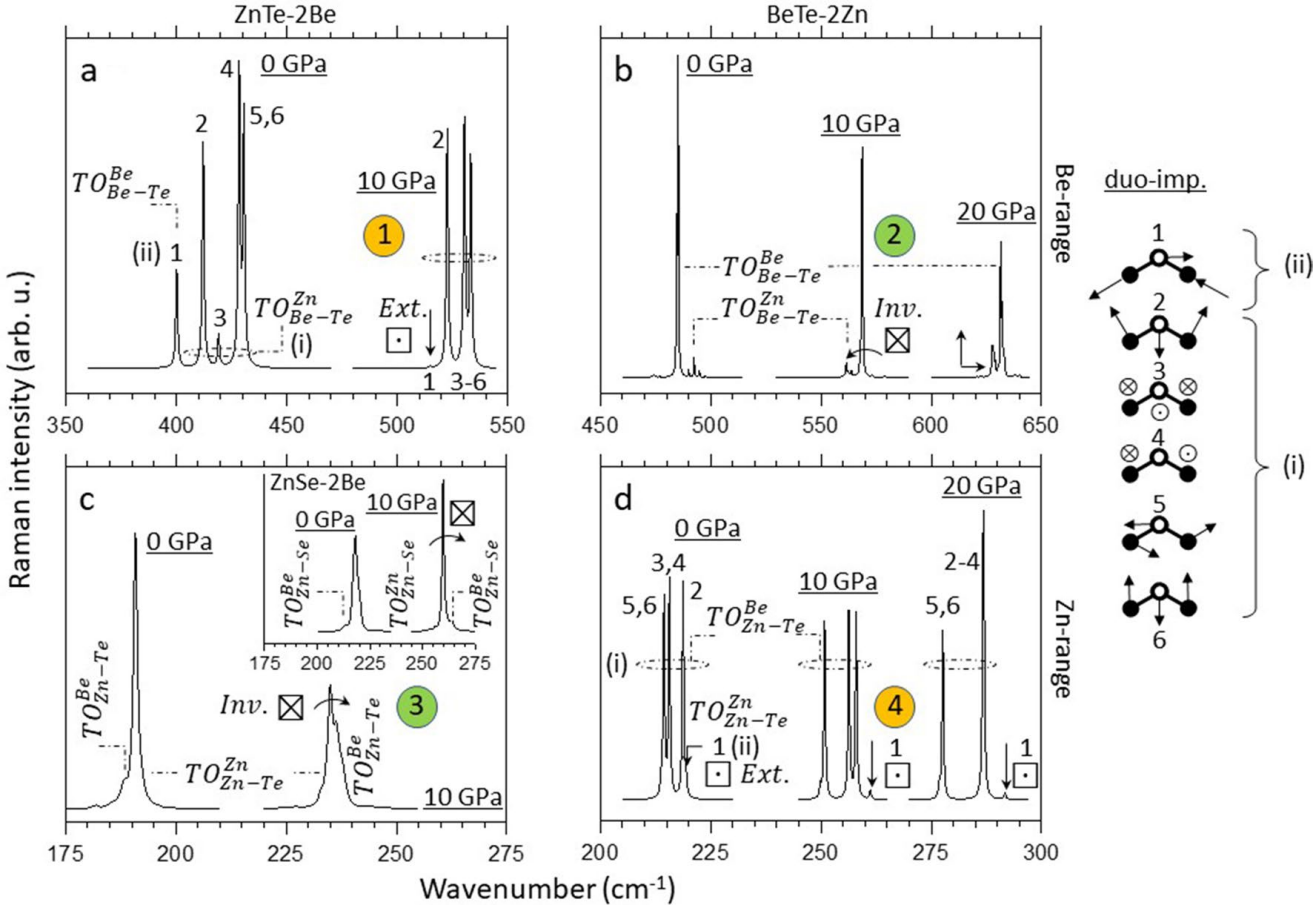
Ab initio (AIMPRO) high-pressure Raman spectra at (x ~ 0,1). Large (216-atom) parent-like zincblende-type supercells containing the prototypical percolation-type impurity motif, i.e., a duo of connected impurities, are used. The six vibration patterns of the impurity-duo (right hand side of the main panel) are regrouped in terms of percolation-type vibrations in foreign (i) and like (ii) environments (in reference to Fig. 1b). **(a–d)** Direct insights into the pressure-induced free/inhibited-coupling regimes per bond taken as the dominant/minor species, using the same symbolic ①-to-④ terminology (together with all related descriptive features, e.g., ⊠ and $Inv.$) as in Fig. 1.

In the Be–Te range, the lower/minor ${TO}_{Be{{-}}Te}^{Be}$ feature becomes overdamped and progressively collapses while converging (curved arrow) onto the upper/dominant ${TO}_{Be{{-}}Te}^{Zn}$ one, until extinction (symbolized ⊡) at the crossing/resonance. This materializes a “phonon exceptional point”, as originally observed with Zn_1−x_Be_x_Se^10^. By analogy, only the upper Be-Te mode is expected to survive the resonance, the lower one is presumably frozen. A direct experimental insight is, however, forbidden since the crossing/resonance, occurring at P_res._ ~ 9 GPa, nearly matches, fortuitously, the zincblende $\to$ cinnabar pressure transition. The (crossing, resonance, extinction) are not due to the latter transition since the three features replicate as such in our ab initio data related to a zincblende-type supercell (referring to ①, Fig. 3). In this line, the experimental observation of a similar “phonon exceptional point” in the zincblende phases of Zn_1−x_Be_x_Se^10^ and Zn_1−x_Be_x_Te (this work) before they adopt the high-pressure rocksalt and Cmcm (via cinnabar) phases, respectively, reveals that the final phase plays no role in the pressure-induced closing process seen in the native phase.

Conversely no exceptional point is experienced in the Zn-Te range. A careful examination reveals that by increasing the pressure from ambient to ~ 1 GPa the minor ${TO}_{Zn-Te}^{Be}$ mode shifts across (symbolized ⊠, curved arrow) the dominant ${TO}_{Zn-Te}^{Zn}$ one. Hence both Zn–Te submodes survive the resonance. Also, once inverted the doublet stabilizes and shifts as a solid body under pressure (from ~ 2.5 GPa onwards). The uniform shift suggests that the two submodes are coupled in some way. However, the coupling is refuted by the lack of transfer of oscillator strength between the two modes. Indeed, each submode exhibits (nearly) the same Raman intensity in the regular (*ante* resonance) and inverted (*post* resonance) doublets. An interesting issue, then, is to elucidate which driving force stabilizes the inverted Zn–Te doublet at high pressure.

The above pending Be–Te and Zn–Te issues at the term of our experimental Zn_1−x_Be_x_Te Raman study at small Be content, together with the missing insight at large Be content, are tackled below by extending to high pressure the ab initio calculations on the Zn_1−x_Be_x_Te supercells at x $\sim$ (0,1). Generally, the AIMPRO (Raman signal, Fig. 3) and SIESTA ($\Gamma$-PhDOS, see Figs. S2, S9 of Supplementary Sects. I, II, related to Zn_30_Be_2_Se and Zn_30_Be_2_Te, respectively) trends are consistent, which gives confidence for the discussion.

At x ~ 0, the abrupt increase in pressure from 0 to 10 GPa generates an inversion (⊠) of the Zn–Te doublet (Fig. 3c). Moreover at 10 GPa a massive transfer of oscillator strength is observed—to a point that the two Zn–Te sub-modes exhibit similar Raman intensities. This proves the «free» Zn–Te coupling. A similar inversion, but preserving the Raman intensities on both sides of the resonance, is likewise evidenced for the Zn–Se doublet of Zn_1−x_Be_x_Se (insert of Fig. 3c), which solves the above mentioned apparent anomaly in the experimental data (Fig. S1). Such ab initio insights give a hint on the basic ingredients (coupling near the resonance, inversion on both sides of it) behind the pressure-induced closing process of the Zn-related Raman doublet in (Zn, Be)-chalcogenides at x ~ 0.

Conversely, in the Be–Te spectral range a similar increase in pressure «inhibits» (⊡) the in-chain stretching along the Be-duo (behind the minor/lower ${TO}_{Be{{-}}Te}^{Zn}$) on the onset of joining the remaining five out-of-chain Be-duo modes (grouped under the upper/dominant ${TO}_{Be{{-}}Te}^{Zn}$) at the resonance (Fig. 3a). Only the out-of-chain modes « survive » the resonance, the in-chain one is «killed», as earlier observed with Zn_1−x_Be_x_Se^10^. At x ~ 1, a phonon exceptional point of the Zn-duo, corresponding to (quasi) degeneracy of its in-chain and out-of-chain modes, is experienced already at 0 GPa (Fig. 3d). Hence the in-chain mode remains frozen from ambient pressure onwards (⊡); only the out-of-chain modes remain active. In contrast, the Be–Te coupling freely develops into an inverted Be–Te doublet at 10 GPa (⊠), then shifted as a (nearly) solid body (up to 20 GPa, Fig. 3b), in echo to the Zn–Te behavior at x ~ 0 (Fig. 3c).

Summarizing, the experimental and ab initio data provide evidence for an effective coupling between the two like percolation-type TO Raman submodes due to a given bond when they are forced into proximity by pressure. Referring, e.g., to x ~ 0, the coupling is apparent in the collapse of the minor (Be–Te) submode (regime ①, Figs. 2, 3) and in the transfert of oscillator strength between the two (Zn–Te) submodes (regime ➂, Fig. 3), different signs that the like submodes actually “see” each other and interact/couple. The coupling must be mechanical in nature since the addressed TO modes with the used Raman setup and ab initio codes consist of purely mechanical vibrations, being deprived of electric field (despite the ionic character of the chemical bonding in a zincblende crystal). Indeed, in the used backscattering geometry the transfered wavector is maximal, and hence not compatible with the propagation of a transverse (photon-like) electric field^10^. Also, in their current versions the AIMPRO and SIESTA codes are not able to treat macroscopic ($\Gamma$-like) electric fields.

Remarkably, the ab initio trends (⊠, ⊡) are consistent with experiment at x $\sim$ 0, and symmetrically replicate at x $\sim$ 0 and 1. We deduce that the free/inhibited-coupling regimes are not bond-related but determined by bond abundance. The strict dominant/minor character is, however, not decisive per se since Zn_1−x_Be_x_Se was shown to exhibit a Be-Se exceptional point at x = 0.52^10^, i.e., even beyond 50 at% Be. This directs the discussion towards the next critical composition, related to the topology of the like bonds of a given species, as to whether they self-connect into (finite or infinite) treelike chains or form a matrix-like continuum, depending on percolation (x $\le$ 0.81, assuming a random Zn $\leftrightarrow$ Be substitution) or non percolation (x $>$ 0.81) of the alternative (Zn-based) bond species^16^, respectively.

A tentative rule for the pressure-induced free/hindered-coupling regimes (⊠, ⊡) of a given Raman doublet enunciates as follows. On approaching the resonance of the two submodes forming a given Raman doublet, an effective mechanical coupling freely develops into a proper inversion of the doublet only when the bond is so dominant that it forms a matrix-like continuum. Otherwise, i.e., when the bond merely self-connects via (finite or infinite) treelike chains, the coupling is undermined by overdamping of the in-chain stretching until its full extinction at the resonance. At this limit a phonon exceptional point, interrupting the crossing process, is experienced. In this case, only the out-of-chain modes survive the resonance, whereas the in-chain one freezes. Note that by application of the given rule to the long species, characterized by a (quasi) degenerated Raman doublet already at ambient pressure (Fig. 3d), it follows that its in-chain mode is permanently inhibited, i.e., at any pressure. This was unsuspected so far.

One remaining step, needed to facilitate discussion, is the joined description of the “free” (⊠) and “inhibited” (⊡) coupling regimes within the same 1D-model—for the sake of consistency with the 1D percolation scheme. The inhibited-coupling regime (referring to ①, Fig. 1), has already been explained on such basis, using Zn_1−x_Be_x_Se as a case study^10^. Hence it is only briefly outlined with Zn_1−x_Be_x_Te (in reference to ①/④). The free-coupling regime (➁/➂, Fig. 1) that was not even suspected with Zn_1−x_Be_x_Se, captures most of the attention.

For clarity we focus on the Be–Te doublet, that is well resolved in experimental and ab initio data both at x ~ 1 (free coupling) and at x ~ 0 (exceptional point, i.e., inhibited coupling)—being clear that the same approach transposes to Zn–Te as well (see Fig. S10 of Supplementary Sect. II). The Be–Te doublet is described in terms of two coupled-damped (mass + spring) harmonic 1D-oscillators along the model of Dolfo and Vigué^24^ adapted for our use with Zn_0.5_Be_0.5_Se^10^. The two 1D-oscillators have identical mass $\mu$ (the reduced mass of a Be–Te bond) but different force constants, noted ${k}_{Be}$ and ${k}_{Zn}$, depending on the (Be- and Zn-like) first-neighbor 1D-environments of Be–Te. The two oscillators are mechanically coupled via a spring-like restoring force (${k}^{{\prime}}$), assumed to be neither pressure nor composition dependent, in a crude approximation. An upper estimate of the coupling frequency ${\omega }^{{\prime}}=\sqrt{k{^{\prime}}/\mu }$—along the procedure used with Zn_1−x_Be_x_Se^10^ (see Fig. S7 of Supplementary Sect. II)—is ~ 50 cm^−1^, i.e., roughly one order of magnitude less than the TO frequency of pure BeTe^26–28^, i.e., ${\omega }_{0}$ ~ 461 cm^−1^. As shown in Fig. 1e, providing a convenient support for the discussion, the coupling (${\beta }^{{\prime}}={\omega }^{{\prime}}/{\omega }_{c}$) vs. overdamping ($\beta =\left|{\gamma }_{Be}-{\gamma }_{Zn}\right|/{\omega }_{c}$) competition dramatically impacts the frequency gap ${\Delta }_{Res}$ between the two normal modes of the coupled system at the resonance (${\omega }_{c}$). We recall that these are due to in-phase (SYM) and out-of-phase (ASYM) displacements of the two masses, with equal magnitude^24^. In the used notation, ${\omega }_{c}$ and (${\gamma }_{Be}$, ${\gamma }_{Zn}$) refer to frequency matching of the bare-uncoupled oscillators at the resonance (${k}_{Be}={k}_{Zn}$) and to the individual phonon dampings (giving the width at half maximum of Raman peaks), respectively.

The free (of overdamping) coupling regime ➁, which cuts the ordinate axis in Fig. 1e, is experienced at x ~ 1 between 0 and 10 GPa. An insight into the transfer of oscillator strength mediated by mechanical coupling on each side of the resonance, which dramatically impacts the Raman intensities, involves modeling of the Raman cross section (RCS) of the coupled system (see “Methods” section). The pressure dependence of the BeTe-like RCS of Zn_1−x_Be_x_Te in absence of (over)damping is shown in Fig. 1c with an arbitray focus on the Zn–Te bond percolation threshold (x = 0.81) presumably separating the free- and inhibited-coupling regimes of Be–Te (see above). Given the ${\omega }^{{\prime}}$ value (~ 50 cm^−1^), the inversion of the Be–Te doublet at 10 GPa leaving the individual Raman intensities unchanged with respect to 0 GPa is achieved by considering the resonance/crossing of the bare-uncoupled oscillators (${k}_{Be}={k}_{Zn}$) at ~ 6 GPa (opposite arrows). The model gives a hint on the repulsion of the coupled oscillators at the resonance (increasing with ${\omega }^{{\prime}}$, shown in Fig. S10 of Supplementary Sect. II) and also on the $\omega^{\prime}$-mediated interplay between the Raman intensities, resulting in the net inversion of the doublet away from the resonance. A symmetrical insight into the free-coupling regime ➂ of the Zn–Te doublet at x = x_Be−Te_ = 0.19, i.e., the Be–Te bond percolation threshold, is provided in Fig. S10 of Supplementary Sect. II, to complete the picture.

Remarkably in the ab initio data at 20 GPa the frequency gap reduces with respect to 10 GPa (Fig. 3b). This manifests a sort of way back towards the resonance, i.e., some mechanical «re-coupling» at high pressure, giving rise, in fact, to a renewed interplay between the Be–Te Raman intensities/frequencies (duo of arrows at 20 GPa). Clearly, the crude extrapolation to high pressure of the basic rule that «the pressure dependence of a Raman doublet reflects a difference in the hardening rates of the local environments of a bond» leads to an anomaly that, once inverted, the Be–Te doublet keeps diverging under pressure, indefinitely. An alternative view is qualitatively depicted in the image of the coupling between the two submodes of a given doublet taking place within a cone-shape frequency domain (see dotted lines, Fig. 1c). The cone shrinks on assumption that the strengthening of the bond force constant progressively outweights the effect of the local environment under pressure. In this case, the two Be–Te submodes naturally converge onto each other at high pressure, which enforces their mechanical coupling away from the resonance.

At x ~ 0, the lower Be–Te mode becomes overdamped and collapses while converging onto the upper one under pressure (Fig. 3a). The overdamping acts against the mechanical coupling that develops between the two oscillators when forced into proximity by pressure. At the resonance the loss (overdamping) achieves maximum to compensate the maximum gain (mechanical coupling) so that the two oscillators, now decoupled, cross (within experimental resolution)—being clear that any effective coupling would otherwise lead to a repulsion^24^. This situation cuts the abscissa axis in Fig. 1e. The unique normal mode at the resonance achieves a compromise between the SYM and ASYM modes in absence of overdamping. Hence, one of the two submodes, the overdamped one, has to be inert. As such, it cannot scatter light any more, which explains its Raman extinction from the resonance onwards. The (doublet closing, Raman extinction, phonon freezing) triptych at the resonance finds a natural explanation within the concept of a “phonon exceptional point” being achieved^10^.

A fair modeling of the Be-Te closing process ① at, e.g., x ~ 0.19 is given in Fig. 1d by setting ${\omega }^{{\prime}}$ to zero (full decoupling by overdamping) in the RCS—then scaling as the imaginary part of the classical relative dielectric function (${\varepsilon }_{r}$) due to two mechanically-independent oscillators (see “Methods” section)—and by considering a linear loss of oscillator strength of the overdamped mode under pressure until extinction at the resonance. As for the undamped oscillator, its oscillator strength is preserved throughout. Only, the value reduces under pressure—impacting the Raman intensity—due to the pressure dependence of its constituent parameters (see Supplementary Sect. II). the same picture/modeling applies to regime ④ of Zn–Te.

## Conclusion

Based on the current high pressure experimental/ab initio Raman study carried out on Zn_1−x_Be_x_Te at x ~ (0,1), a basic rule emerges regarding the achievement, or not, of a phonon exceptional point on closing of the percolation-type Raman doublets of zincblende mixed crystals, as to whether the mechanical coupling is inhibited at the resonance or freely develops into an inverted doublet, respectively, with phenomenological modeling in support. The given rule—detailed in the main text—points towards a pivotal role of the bond percolation thresholds, i.e., of the mesostructure, regarding the pressure dependence of the vibrational properties of a zincblende-type mixed crystal. Besides its fundamental interest, the given rule opens up promising prospects for applications. In fact, the inhibition/release of bond vibrations in mixed crystals in the overdamped/free coupling regimes, depending on composition and pressure, offers various versatile off/on phononic switches at the mesoscopic scale, yet unexploited. On the fundamental side, one possible perspective to this work is to investigate whether the currently established percolation picture for the pressure dependence of the vibrational properties of common semiconductor mixed crystals with the high-symmetry zincblende structure can be transposed, or not, to less symmetrical crystal structures. A prerequisite, however, is to formalize the corresponding percolation scheme(s) at ambient pressure.

## Methods

This Section provides details concerning the samples, Raman setup, (AIMPRO) ab initio method and linear dielectric approach used to acquire, support and formalize the discussed data in the main manuscript. Complementary experimental and numerical data, obtained by high-pressure Raman scattering, high-pressure X-ray diffraction, solid-state nuclear magnetic resonance measurements and (SIESTA) ab initio calculations are reported in the Supplementary Sects. I and II.

### Samples

High-quality Zn_1−x_Be_x_Te single crystals needed to perform high-pressure Raman and X-ray diffraction measurements were grown from the melt by mixing high-purity ZnTe (5 N quality, i.e., 99.9995) and Be (2 N quality, i.e., 99.5) materials using the high-pressure Bridgman method^15^, at various Be contents (x = 0.045, 0.11, 0.14, 0.21) spanning the accessible composition domain with this technique (x $\le$ 0.21). In their raw form the samples appear as small ~ 2 mm^3^ crystal pieces taken from the original cylindrical ingots. They all exhibit the ZnTe-like zincblende structure at ambient pressure, checked by X-ray diffraction, further used to estimate the composition x assuming a linear dependence of the lattice constant.

### High-pressure Raman measurements

The high-pressure Zn_0.86_Be_0.14_Te Raman measurements were done by using the 632.8 nm helium–neon laser line in backscattering on a single crystal (~ 50 $\times$ 50 $\times$ 50 μm^3^) inserted into a 150 μm in diameter cylindral cavity drilled by spark erosion through a stainless-steel gasket preindented to 80 μm, taken between large ultra-low fluorescence diamonds (400 μm in culet, with Boehler–Almax design) of a membrane Chervin type diamond anvil cell^32^. Methanol/ethanol/distilled-water in volumic proportion 16:3:1 was used as the pressure transmitting medium since this mixture remains hydrostatic up to ~ 10.5 GPa^33^, conveniently covering the zincblende-structure domain of our crystals—of central interest. The pressure inside the cavity was measured via ruby chips placed next to the crystal using the fluorescence linear scale^34^. The discussed Raman trends in the upstroke regime (pressure increase) are reversible in the downstroke regime (pressure decrease)—with a slight hysteresis, though, corresponding to a delay of ~ 2 GPa in the crystal phase transitions—and hence intrinsic to the mixed crystals.

### Ab initio high-pressure Raman spectra

The high-pressure pure-TO Raman spectra of Zn_1−x_Be_x_Te taken in its dilute/parent limits (x ~ 0,1) are calculated using the formula given by de Gironcoli^35^ by applying the AIMPRO code^21,22^ operated within the density functional theory (DFT) and Pseudopotential approximation^36^, along the local density approximation for the exchange–correlation potential, using large (216-atom) supercells with zincblende structure containing the prototypical percolation-type impurity motif, i.e., a duo of impuritys connected via an unvariant Te atom. The 3*d* Zn electrons were considered as valence electrons and the Brillouin-zone was sampled at the MP-2^3^ special k-points^37^. The Raman calculations are done after full relaxation of the supercells, i.e., of the lattice constant—determined via the third-order Birch–Murnaghan equation of state^38^—and of the internal atom coordinates. By applying a step increase in pressure from ambient to 10 GPa to a reference pure-ZnTe (to 20 GPa in pure-BeTe) supercell the TO mode is upward shifted by ~ 50 cm^−1^ (~ 144 cm^−1^) consistent with experimental findings in this work (other DFT calculations^39^), resulting in a positive test.

### Phenomenological modeling of (high-pressure) Raman spectra

The Raman cross section (RCS) of the percolation-type purely-mechanical TO Raman doublet of a given bond of Zn_1−x_Be_x_Te is calculated by applying the linear dielectric formalism of Hon and Faust^40^ to a system of two mechanically-coupled 1D-harmonic oscillators in absence of overdamping. A crude approximation of the RCS by taking the imaginary part of the relative dielectric function (${\varepsilon }_{r}$) of the coupled system—derived by adding a virtual coulombian coupling between the 1D-oscillators via a common electric field ($E$, to be removed at a later stage) on top of their mechanical coupling ($k^{\prime}$)—while successful in absence of mechanical coupling, generates a non-physical antiresonance in case of a finite mechanical coupling, and hence is not exploitable for our use. Therefore, a strict calculation of the RCS is done, searching for its asymptotic limit at ${\varepsilon }_{r}\to \infty$ (achieved for $E$ = 0, restoring the purely-mechanical character). Detail is given in the Supplementary Sect. II.

## Supplementary Information

Supplementary Information.

## Data Availability

The reported experimental (Raman, high-pressure X-ray diffraction, nuclear magnetic resonance) and ab initio (AIMPRO and SIESTA codes) data in this work are available upon request to the corresponding author.
