# Supplementary material for: Exceptional phonon point versus free phonon coupling in Zn1−xBexTe under pressure: an experimental and ab initio Raman study
Source: Sci Rep. 2022 Jan 14;12:753. doi: 10.1038/s41598-022-04815-w (PMC8760325; doi:10.1038/s41598-022-04815-w)
Supplement: Supplementary file 1 — Supplementary Information. [file 41598_2022_4815_MOESM1_ESM.docx]

**Supplementary Information**

**Exceptional phonon point versus free phonon coupling in Zn_1-x_Be_x_Te under pressure: an experimental and *ab initio* Raman study**

M. B. Shoker, T. Alhaddad, O. Pagès, V. J. B. Torres, A.V. Postnikov, A. Polian, R. Hajj Hussein, G. K. Pradhan, C. Narayana, C. Gardiennet, G. Kervern, L. Nataf, S. Ravy, J.-P. Itié, K. Strzałkowski, A. Marasek, and F. Firszt

In this annex we report additional information concerning the (Zn,Be)-chalcogenide mixed crystals. Sec. I relates to the pure-TO Raman signal of Zn_1‑x_Be_x_Se depending on pressure, studied both experimentally (Sec. I.1) and using the *ab initio* SIESTA code (Sec. I.2), with special attention to the Zn‑Se spectral range. Sec. II is concerned with Zn_1‑x_Be_x_Te, of central interest in this manuscript, covering both its structural and vibrational properties. The pressure dependence of the crystal structure of Zn_0.86_Be_0.14_Te studied in Sec. II.1 constitutes a prerequisite to its high-pressure Raman study in the main text. An attempt to achieve a quantitative experimental insight on the nature of the Zn$\leftrightarrow$Be atom substitution by applying solid-state nuclear magnetic resonance to the ^125^Te-unvariant, though unsuccessful, is presented in Sec. II.2 for the sake of completeness. Sec. II.3 reports additional Zn_1‑x_Be_x_Te Raman data completing and supporting those discussed in the main text. The pure-TO Raman signals of the current bulk crystals are compared with those of earlier studied epitaxial layers with similar composition in Sec. II.3.a. This sheds light onto the nature of the Zn$\leftrightarrow$Be atom substitution in the studied Zn_1‑x_Be_x_Te bulk crystals, by analogy. An experimental insight into the amount of mechanical coupling ($\omega'$) between the two Be-Te sub-oscillators is achieved in Sec. II.3.b by implementing the model of Dolfo and Vigué^24^ on TO and LO Raman frequencies earlier gained from a Zn_~0.5_Be_~0.5_Te epitaxial layer. The experimental high-pressure Raman study of Zn_0.89_Be_0.11_Te done in Sec. II.3.c helps to ascertain the reproducible and alloy-intrinsic character of the discussed Zn_0.86_Be_0.14_Te features in the main text. Besides, to check the reproducibility of all discussed *ab initio* trends we replicate with the SIESTA code in Sec. II.3.d the AIMPRO Zn_1-x_Be_x_Te phonon calculations done in the main text. Last, in Sec. II.3.e we outline a linear dielectric approach, operated within the formalism of Hon and Faust,^40^ to calculate the Raman cross section of the percolation-type TO Raman doublet of a given bond in a zincblende-type mixed crystal, modeled as a mechanically- and electrically-coupled system of two harmonic 1D-oscillators, in the spirit of a recent approach by Dolfo and Vigué^24^.

1. **High-pressure Raman spectra of Zn_1-x_Be_x_Se**

I.1. Experimental study at moderate Be content

A selection of powder high-pressure Raman spectra taken in the native zincblende phase of various Zn_1‑x_Be_x_Se single crystals (x=0.16, 0.24 and 0.52) spanning the composition domain up to the highest achievable Be content (x$\leq$0.52) with the used growth Bridgman technique is shown in Fig. S1 (inserts a, b and c, respectively). Only a small series of Raman spectra is shown for Zn_0.76_Be_0.24_Se because their pressure dependence, similar in every respect to those currently reported for Zn_0.84_Be_0.16_Se and Zn_0.48_Be_0.52_Se, is detailed in Ref. 17 (Fig. 1 therein). Alternative high-pressure Raman spectra of the studied crystals can also be found in Refs. S1-S3, with an emphasis on the Be-Se spectral range or in relation to the Zn-Se phonon-polaritons – corresponding to the polar variant (with mixed mechanical/electrical character) of TO modes accessible by near-forward Raman scattering. In the current study the emphasis is shifted to the regular (purely-mechanical) Zn-Se TO modes, observed in the conventional backward scattering geometry. The currently reported data were acquired with the 532.0 nm laser line using a similar high-pressure Raman experimental setup (see Methods). The considered pressure domain is comparable to that explored with Zn_1‑x_Be_x_Te in the main text, to facilitate comparison.

At ambient pressure the ZnSe-like TO Raman signal exhibits a complex lineshape – that basically replicates at all studied compositions – due to a severe contamination of the discrete zone-center TO mode by various zone-edge two-phonon acoustic continua (2TA, TA-LA) that emerge nearby on its low frequency side.^13^ By increasing pressure the TO mode is upward shifted while the TA-LA and 2TA bands keep vibrating at a similar frequency or are downward shifted, respectively (see main text)^30,31,17^. This results in a clear resolution of all individual features/bands at high pressure (~13 GPa), offering a clarified insight into the ZnSe-like TO vibration pattern. The Raman intensity ratio between the lower and upper intermediate features, framed by the low-frequency 2TA band and the high-frequency ${LO}_{Zn-Se}$ mode, roughly scales as $x/\left( 1-x \right)$, *i.e.*, in proportion to the individual fractions of 1D-oscillators behind the $\left\{ {TO}_{Zn-Se}^{Be},{TO}_{Zn-Se}^{Zn} \right\}$ percolation-type Zn-Se Raman doublet (refer to the terms in square brackets in Fig. 1). This cannot be merely fortuitous, suggesting that the lower and upper features in question basically relate to the above-cited doublet – in this order. The pressure-dependence of the two features (dotted lines in Fig. S1) reflect an apparent divergence of the ZnSe-like TO doublets under pressure, at any composition, contradicting the expected convergence in our recent classification of mixed crystals in terms of closing/opening-type systems under pressure^10^. Now, the lower ${TO}_{Zn-Se}^{Be}$ mode remains presumably contaminated by the spurious TA-LA band on account that it remains pined at a nearly fixed frequency under pressure, in contrast with the upper ${TO}_{Zn-Se}^{Zn}$ one that is upward shifted, as expected^30,31^. We conclude that the current experimental insight with Zn_1‑x_Be_x_Se is hampered by the TA-LA/${TO}_{Zn-Se}^{Be}$ interference, and, hence, not reliable.

I.2. *Ab initio* (SIESTA code) study in the (Be,Zn)-dilute limits

A reliable insight into the pressure dependence of the ZnSe-like TO doublet of Zn_1-x_Be_x_Se is presented in the main text. We performed *ab initio* (AIMPRO) calculations of the high-pressure TO Raman spectra using a large Zn_106_Be_2_Se_108_ supercell with zincblende structure containing the prototypical Be-duo percolation-type impurity motif. In contradiction with experimental findings (see above), the AIMPRO data (inset, Fig. 3c) show a pressure-induced closing of the ZnSe-like TO doublet, conforming with predictions^10^. Remarkably, the closing develops into a proper inversion of the doublet at high pressure (10 GPa).

Similar (SIESTA) calculations of the $\Gamma$-projected phonon density of states ($\Gamma$-like PhDOS) – that assimilates with the pure-TO Raman spectrum in a crude approximation – related to a Zn_30_Be_2_Se_32_ zincblende-type supercell depending on pressure, using the method and basis of pseudopotentials set up in Refs. 30 and 17 are displayed in Fig. S2. The reported SIESTA data distinguish between the $\Gamma$-like PhDOS normalized per Zn depending on whether Zn connects to the Se atom bridging the Be-duo, or not, in reference to Zn atoms vibrating in Be- (thick curve) and Zn-like (thin curve) environments, respectively. At ambient pressure (Fig. S2a) the Zn atoms vibrate a (slightly) lower frequency close to the Be-duo than away from it (by less than 0.5 cm^-1^, referring to the dominant $\Gamma$-like PhDOS feature), and the trend is reversed at 18 GPa (Fig. S2b). This nicely recollects with the AIMPRO findings (see main text).

The SIESTA data are further useful to follow the six vibrational modes related to the Be-duo in their pressure dependence beyond the 10 GPa pressure studied in earlier work.^17^ The individual modes are labeled along the same terminology (the global i-ii and detailed 1-6 ones) as in Fig. 3, for the sake of consistency, and likewise regrouped (ovals) under the corresponding $\left\{ {TO}_{Zn-Se}^{Be},{TO}_{Zn-Se}^{Zn} \right\}$ features in the overlying percolation scheme. Retrospectively (in the light of Ref. 10), an exceptional phonon point was achieved for the Be-duo at 10 GPa, signed by extinction of its in-chain mode (see Fig. 3 of Ref. 17). The Be-Se signal at 18 GPa (current data) replicates as such that earlier observed at 10 GPa – if we omit an overall upward shift due to the pressure increase. This confirms our view^10^ that the Raman signal remains stable in form under pressure once an exceptional phonon point has been achieved – supporting the trend modeled in Fig. 1d.

1. **Zn_1-x_Be_x_Te**

II.1. High-pressure X-ray diffraction

X-ray diffraction measurements were performed at the CRISTAL beamline of the SOLEIL synchrotron using the 0.485 $Å$ radiation on finely grounded powder. The same Chervin type diamond anvil cell as for the Raman measurements was used. Only, as the structural phase transitions of Zn_0.86_Be_0.14_Te were explored up to pressures exceeding by far the hydrostatic limit (~10 GPa) of the used methanol/ethanol/distilled-water mixture in the Zn_0.86_Be_0.14_Te high-pressure Raman measurements, neon was preferred as the pressure transmitting medium, and further used as a sensitive marker of the pressure inside the sample cavity^S4^. The x-ray beam was focused onto a ~40 mm in diameter sample spot. The diffracted light was collected onto a plane detector positioned at ~33 cm at the rear of the sample, and converted into a proper intensity versus $2\theta$ plot using the FIT2D^S5^ software. The peaks fitting and unit cell fitting was carried out using the software DATLAB (kindly provided by K. Syassen, Max Planck Institut für Festkörperphysyk, Stuttgart, Germany).

Representative series of high-pressure x-ray diffractions patterns of Zn_0.86_Be_0.14_Te taken from ambient pressure up to 24 GPa at the upstroke and downstroke are shown in Fig. S3a and S3b, respectively. The emergence and disappearance of crystal phases are emphasized.

Basically, Zn_0.86_Be_0.14_Te exhibits the same successive zincblende, cinnabar and Cmcm crystal phases as ZnTe under pressure. Only, ZnTe hardens with Be incorporation (see main text), resulting in an upward shift of all critical pressure transitions^19,20,25,S6^, presently detected at 12.7 GPa and 14.6 GPa in the upstroke regime. The transitions reversibly occur in the downstroke regime with a slight hysteresis, the critical pressures being displaced to lower values by ~2 GPa. The lattice constants pressure dependence at the upstroke (filled symbols) and downstroke regimes (hollow symbols) are detailed in Fig. S4. Out of these, the zincblende data are eventually used to derive the Zn_0.86_Be_0.14_Te bulk modulus at ambient pressure $B_{0}$, with an average value of 50.8 GPa framed between the extreme ones of 48.9 GPa and 54.2 GPa, as estimated via a second-order Birch-Murnaghan equation^38^ ($B_{0}^{'}$=4)^S6^. Such $B_{0}$ value for Zn_0.86_Be_0.14_Te fits into the linear dependence between the ZnTe (50.5 GPa, Ref. S7) and BeTe (67.0 GPa, Ref. S8) parent values, within error bars.

II.2. Solid-state nuclear magnetic resonance

Solid-state nuclear magnetic resonance (NMR) measurements of the chemical shift due to the unvariant atom species (C in this case) of zincblende A_1-x_B_x_C mixed crystals demonstrated sensitivity to the local environment defined at the first-neighbor scale of the probed species, out of five possible variants at any composition x, corresponding to as many C-centered tetrahedral clusters with zero to four A atoms at the vertices, completed by B^S9^. The relative abundance of the various clusters at a given composition of the crystal is measurable via the integrals of the related NMR peaks, to be compared with the theoretical distribution given by the binomial Bernoulli’s distribution. In the ideal case of agreement between the experimental and Bernoulli’s distributions, one may safely conclude that the A$\leftrightarrow$B substitution is random in the studied crystal. A general form of the binomial Bernoulli’s distribution equipped with a relevant order parameter ($\kappa$) varying on a positive scale of 0 (random distribution) to 1 (full clustering, corresponding to phase separation), and also negative values (anticlustering, down to the limit of -1 only achievable at x=0.5, corresponding to alternation of A and B on the face centered cubic substitution sublattice), derived by Verleur and Barker^9^, can further be used to estimate any deviation from the ideal random substitution towards clustering or anticlustering, as recently done with Zn_1‑x_Cd_x_Se^10^. One prerequisite, however, is a one-to-one correlation between the observed NMR peaks and the (at most) five possible C-centered tetrahedral units of the crystal.

A classical quantitative one-dimensional ^125^Te NMR signal of a powdered Zn_0.89_Be_0.11_Te sample is shown in Fig. S5. It was obtained in a saturation recovery experiment corresponding to five times the longitudinal relaxation time T1~290 s (cycled over 60h) using a Bruker Avance III 600 MHz spectrometer (14T) equipped with a Bruker 1.3 mm triple resonance Magic Angle Spinning probe. The ^125^Te NMR signal measured in similar condition (T1~400 s) with a ZnTe powder used for external calibration of the ^125^Te chemical, resonating at -888 ppm consistently with existing measurements in the literature^S10^, is added for reference purpose.

As observed with Zn_1-x_Cd_x_Te^S9^, the ZnTe peak falls within the spectral domain covered by the Zn_0.89_Be_0.11_Te ^125^Te NMR signal. This decomposes into five broad peaks, located at -855 ppm, -875 ppm, -895 ppm, -915 ppm and -945 ppm. However, only two contributions are expected based on the binomial Bernoulli’s distribution in case of a random substitution (inset of Fig. S5). Such pronounced deviation remains obscure at present since Zn_1-x_Be_x_Te epitaxial layers were found to exhibit a (quasi) random Zn$\leftrightarrow$Be substitution by Raman scattering (see below)^7^. Our present view is that the unusually large contrast between the Zn-Te and Be-Te bond physical properties (see main text) is challenging for any one-to-one correspondence between the NMR peaks and the elementary Te-centered tetrahedra forming Zn_1‑x_Be_x_Te. A more advanced NMR study covering well-spanned Be contents (undermined by the highest achievable limit of ~21 at.%), falling beyond the scope of this work, would be needed in view to establish such reliable correspondence, if any.

II.3. Raman scattering

II.3.a. Epitaxial layers vs. bulk ingots : nature of the Zn$\leftrightarrow$Be substitution process

An alternative approach is to rely on the existing Raman insight into the nature of the Zn$\leftrightarrow$Be substitution in Zn_1‑x_Be_x_Te gained from the intensity ratio between the two like TO submodes forming the Zn-Te and Be-Te percolation doublets of epitaxial layers (deposited on InP), that revealed a (quasi) random Zn$\leftrightarrow$Be substitution process^7^, and then to infer information about our bulk crystals by analogy/comparison.

For doing so, we superimpose in Fig. S6 two series of corresponding Raman spectra taken with the close 632.8 (present data referring to bulk crystals) and 647.1 nm (ancient data referring to epitaxial layers, taken from Ref. 7) laser lines in the backscattering geometry operated at normal incidence/detection along the (110)-cleaved and -edge crystal faces of Zn_1‑x_Be_x_Te bulk and epitaxial crystals with close compositions (x~0.11, ~0.14, within 1.5%), respectively. The LO modes are forbidden in this geometry, offering a clear insight into both the Zn-Te and Be-Te TO doublets (allowed).

Remarkably, the Zn-Te and Be-Te Raman signals of the two sample series do match almost perfectly within thickness of curves, considering not only the frequencies and intensities of all individual optic modes (four in total) but also their widths at half maximum. The only difference is concerned with the larger/smaller activation of the acoustic features/bands in the Raman spectra of the epitaxial/bulk crystals, not impacting our discussion centered on the optic modes. Hence, by analogy with epitaxial layers^7^, we conclude to a random Zn$\leftrightarrow$Be substitution in our current Zn_1‑x_Be_x_Te bulk crystals.

II.3.b. Epitaxial layers: (TO,LO)-insight into the Be-Te mechanical coupling ($\omega^{'}$)

A crude insight into the mechanical coupling represented by its characteristic frequency ($\omega^{'}$) between the two mechanical 1D-oscillators forming the percolation-type Be-Te doublet of purely-mechanical TO modes $\left\{ {TO}_{Be-Te}^{Be},{TO}_{Be-Te}^{Zn} \right\}$ of Zn_1‑x_Be_x_Te, as described along the model worked out by Dolfo and Vigué^24^ recently adapted for our use with Zn_1-x_Be_x_Se^10^, is achieved hereafter by following the same procedure as originally implemented with Zn_1‑x_Be_x_Se^10^.

More precisely, using the two observed Be-Te TO frequencies as input parameters, $\omega^{'}$ is progressively varied, impacting notably the coupled-LO frequencies (and also the coupled-TO ones), as shown in Fig. S7, until a perfect matching is achieved between the experimental frequency of the lower/overdamped ${LO}_{Be-Te}^{-}$ mode – used as the most relevant marker^10^ – and the corresponding theoretical value. The involved $\omega^{'}$ value at this limit is the desired one.

The above approach is subject to a number of technical constraints. The two mechanically-coupled 1D-oscillators (TO-like) are treated on equal footing in the model of Dolfo and Vigué^24^, by construction. Therefore the approach has to be implemented at x~0.5, corresponding to equal representation of the two percolation-type Be-Te sub-oscillators in the crystal (refer to the terms within square brackets in Fig. 1). As already mentioned, such Be content in Zn_1‑x_Be_x_Te can be achieved only by epitaxy^26,27^. An additional interest for using epitaxial layers is that separate insights into the LO and TO frequencies can be achieved by implementing the backscattering geometry at normal incidence/detection along the (001)-growth and (110)-edge crystal axis, respectively. A pure-LO access is not as straightforward using bulk crystals.

The used input experimental $\left\{ {TO}_{Be-Te}^{Be},{{LO}_{Be-Te}^{-},TO}_{Be-Te}^{Zn},{LO}_{Be-Te}^{+} \right\}$ Raman frequencies (marked by arrows in Fig. S7) are taken from the pure-TO and pure-LO Raman spectra of a Zn_0.48_Be_0.52_Te epitaxial layer (shown in Fig. 3 of Ref. 26) with composition close to the targeted Be content (50 at.%). The experimental frequency of the minor/overdamped ${LO}_{Be-Te}^{-}$ Raman feature, marred by a large error bar (dashed area), matches the theoretical value for $\omega^{'}$=25$\pm$25 cm^-1^. This suggests that the two $\left\{ {TO}_{Be-Te}^{Be},{TO}_{Be-Te}^{Zn} \right\}$ oscillators are quasi decoupled/independent at ambient pressure. However, as the two mechanical modes couple when forced into proximity by pressure (as evidenced by experimental and *ab initio* data in the main text), we have to consider a finite $\omega^{'}$ value in any attempt to model the pressure-induced $\left\{ {TO}_{Be-Te}^{Be},{TO}_{Be-Te}^{Zn} \right\}$ closing process apparent in the (experimental and *ab initio*) Raman spectra. We take $\omega^{'}$=50 cm^-1^ – representing roughly 10% of the TO mode in the pure BeTe compound^26^ – as an upper estimate (a similar scaling applied for the Be-Se coupling in Zn_1-x_Be_x_Se – Ref. 10), considered to be both pressure- and composition-independent, in a crude approximation.

II.3.c. Experimental high-pressure Raman spectra – Zn_0.89_Be_0.11_Te

For the sake of reproducibility of the experimental high-pressure Raman data, we report in Fig. S8 a selection of Zn_0.89_Be_0.11_Te high-pressure Raman spectra taken in the upstroke regime, to parallel with the Zn_0.86_Be_0.14_Te series (Fig. 2). Part of the low-frequency signal, of little interest for our concern, was omitted for clarity (the Zn_0.86_Be_0.14_Te data being already displayed in whole). Both the pressure-induced closing processes (curved arrows) ending up either in an exceptional phonon point ($⊡$) being achieved at the resonance in the Be-Te spectral range – which occurs slightly *ante* the zincblende-to-cinnabar transition detected at ~11 GPa, or in an actual inversion ($⊠$) of the percolation-type doublet in the Zn‑Te spectral range – visible from 2.3 GPa onwards, are observed in the two series of spectra. We have checked that the trends repeat in the downstroke regime, suggesting that they are alloy-intrinsic.

II.3.d. *Ab initio* (SIESTA) high-pressure Raman spectra – Zn_1-x_Be_x_Te at x~0

For the sake of reproducibility of the *ab initio* high-pressure Raman data, we repeat with the SIESTA code in Fig. S9 the AIMPRO phonon calculations done at x~0 in Fig. 3. Only, as already mentioned, while the AIMPRO data consist of the Raman spectra of purely mechanical TO modes, the SIESTA ones refer to the equivalent (in a crude approximation) $\Gamma$-projected phonon density of states ($\Gamma$-like PhDOS) per atom. Besides, a smaller (64-atom) zincblende-type Zn_30_Be_2_Te_32_ supercell (sufficiently converged in size for our use, Refs. 17 and 30) is used with SIESTA – on account of the prohibitive computation time given our computing facilities.

Electronic structure of 62–atomic cubic supercells and hence resulting equilibrium crystal structures of these supercells have been calculated using the SIESTA method^S11^ which applies atom-centered strictly localized basis function. The calculations under pressure have been done by applying the target pressure in the process of the structure relaxation. In many aspects the calculation setup resembles that of Ref. 30, for (Zn,Be)Se pseudobinary alloy. Local density approximation (LDA) was used for the exchange-correlation. The atomic configuration for the construction of Te pseudopotential was the following [cutoff radii in Bohr are specified after each electronic shell: 5*s*^2^(2.40); 5*p*^4^(2.58); 4*d*^10^(1.80); 4*f*^0^(2.49)]; therefore the Te4*d* states were explicitly included in the calculation as the valence ones. The basis sets were of “double-zeta with polarization” quality; the “PAO.EnergyShift” parameter was set to 0.01 Ry, which resulted in maximal extensions of the basis functions of 6.35, 6.61 and 6.35 Bohr for Zn, Be and Te, correspondingly. The “MeshCutoff” parameter, responsible for the fineness of the spatial grid in the representation of the charge density, was fixed at 400 Ry. The **k**-grid for Brillouin zone integration was constructed with effective real-space cutoff (see Ref. S12) of 30 Å. The frequencies and eigenvalues of $\Gamma$-phonons have been calculated within the standard procedure within SIESTA, by applying small individual displacements to all the atoms in the supercell and collecting the force constants, with subsequent diagonalization of the Hessian.

As ideally expected the $\Gamma$-like PhDOS per Be and Zn atoms consistently replicate the AIMPRO trends at x~0, corresponding to an exceptional phonon point ($⊡$) being achieved at 10 GPa in the Be-Te spectral range – reflected by the (quasi) extinction of the in-chain Be-duo mode, together with the premises for an inversion ($⊠$) of the Zn-Te doublet when changing from ambient pressure to 10 GPa. Though the Zn-Te inversion is not as obvious in the Zn-like PhDOS (SIESTA code) as in the actual Raman spectra (AIMPRO code, Fig. 3c), the SIESTA and AIMPRO data are generally consistent at x~0.

Similar SIESTA calculations done at x~1 on a Zn_2_Be_30_Te_32_ supercell (obtained by inverting Zn and Be in the original Zn_30_Be_2_Te_32_ one) are not conclusive (not shown), on account of the very compact $\Gamma$-like PhDOS per Be and Zn atoms, at both ambient pressure and 10 GPa. With this respect, resorting to the AIMPRO code has truly been rewarding/decisive (Figs. 3b and 3d).

II.3.e. TO-like Raman cross section of a system of two mechanically-coupled oscillators

Recently we adapted the formalism used by Dolfo and Vigué^24^ to determine the frequencies of a coupled/overdamped system of two purely-mechanic harmonic (mass + spring) 1D-oscillators, into a “polar” version applying to oscillators with mixed electric-mechanic character. This was needed to address the coupling vs. overdamping issue raised by Dolfo and Vigué in relation with neighboring TO (non-polar, *i.e.*, purely-mechanic) and LO (polar, *i.e.*, with a mixed mechanic-electric character) modes of a zincblende crystal, using Zn_0.5_Be_0.5_Se as a case system.^10^ In the equations of motion per oscillator the introduction of a conventional friction force besides the spring-like restoring force and the Coulomb one resulted in a non-quartic form (likely to provide a paired solution of frequencies, as ideally expected for two coupled oscillators) for the secular equation derived by setting to zero the determinant of the dynamical matrix. The quartic form was restored by Dolfo and Vigué^24^ by substituting for the real/conventional friction force an imaginary one (not in-phase with displacement, introducing a concept of anelastic damping). By doing so the (real parts of the) eigenfrequencies of the coupled systems of TO and LO modes are not only dependent on the (reduced masses, spring-like force constants, effective dynamic charge) of the chemical bonds, but also on the spring-like restoring force constant materializing the mechanical coupling between oscillators, and on the competing damping term. The “exceptional phonon point” experienced under pressure by the Be-Te TO Raman doublet of Zn_0.5_Be_0.5_Se was explained along this line in Ref. 10, with phenomenological modeling in support.

Our current approach is inspired from that of Dolfo and Vigué but follows a different route in that the overdamping is not an issue. Indeed we are interested in the Raman cross section of two undamped harmonic oscillators which mechanically couple when forced into proximity by pressure. More precisely, we aim at examining how the free (of overdamping) mechanical coupling impacts the Raman (frequencies, intensities) on both sides of the resonance (corresponding to frequency matching of the bare-uncoupled oscillators), with special attention to TO modes. A practical case refers to the two 1D-oscillators behind the percolation Be-Te TO Raman doublet of Zn_1-x_Be_x_Te (Fig. 1c).

One basic difference with the approach of Dolfo and Vigué is that the conventional form of the friction force can be used as such in the force assessment per oscillator for what regards the calculation of the Raman cross section, whereas this form was disruptive with respect to the secular equation. In fact, the friction force is required to provide an imaginary character to the coupled system of dynamic equations, the *sine qua non* condition to implement the Hon and Faust formalism^S13^, as apparent below. More prosaically, the damping term is also needed to monitor the full width at half maximum of individual Raman features. Though our attention is centered on the (purely-mechanical, *i.e.*, non polar) TO modes, we cannot escape a more general calculation of the Raman cross section due to the (mechanic-electric polar) LO modes – the reason is made clear at a later stage – before its simplification into a TO version.

At this stage it is useful to recall briefly to which extent the TO and LO modes detected in a conventional Raman experiment done in the backscattering geometry resemble/differ in nature. For doing so we focus on a pure zincblende compound, *i.e*., a mono-oscillator (TO,LO) system, for clarity. Generally, as an optical technique, Raman scattering operates near the center $\Gamma$ (q~0) of the Brillouin zone. At this limit the optic modes consist of out-phase vibrations of the intercalated cation and anion face centered cubic sublattices, both taken as quasi rigid ($\lambda\to\infty$). The relevant 1D-oscillator behind such lattice vibration in a zincblende compound basically refers to the stretching of the chemical bond, abbreviated $u$ from now on, characterizing both the TO and LO modes. For the LO mode, an additional electric character is justified on account that the chemical bonding is partially ionic in a zincblende-type (I-VII, II-VI, III-V) semiconductor. The electric character disappears for a TO mode detected in the backscattering geometry – as in the present case – because the transferred wavevector is maximum in this geometry, and, hence, falls far away from the (quasi vertical) dispersion of a photon, *i.e*., a pure transverse electric field. In this case the transverse electric field which is likely to accompany a TO mode in a polar crystal cannot propagate in fact. The resulting TO mode, deprived of electric field, hence reduces to a purely-mechanical vibration. The LO mode is not subject to the above photon-like restriction because its accompanying electric field $E$ is longitudinal in character. More detail is given in Ref. S13. Note also that the current versions of the AIMPRO and SIESTA *ab initio* codes fail to take into account the macroscopic ($\lambda\to\infty$) electric field of the $\Gamma$-like optical modes, due to the microscopic character of such approaches. Summarizing, the LO mode is jointly characterized by ($u$,$E$) whereas the TO mode by ($u$) only.

A ternary mixed crystal consists of a complex multi-oscillator system, *i.e*., four in fact $\left\{ {{TO}_{Zn-Te}^{Be},TO}_{Zn-Te}^{Be},{TO}_{Be-Te}^{Be},{TO}_{Be-Te}^{Zn} \right\}$ within the percolation scheme, referring to TO modes. For the current study we focus on the Be-Te doublet, as a case study. This can be safely considered to vibrate independently from the Zn-Te doublet, in a first approximation, owing to the large frequency gap between the two doublets (~200 cm^-1^). Below we calculate the Raman cross section of the percolation-type Be-Te Raman doublet of Zn_1‑x_Be_x_Te when the two submodes, forced into proximity by pressure, do mechanically couple.

We recall that the percolation-type Be-Te TO-like Raman doublet distinguishes between Be-Te bonds depending on whether they vibrate in like ($u$_1_) or foreign ($u$_2_) environments. As for the related LO doublet, the involved electric field $E$, macroscopic in character, is the same for both sub-oscillators. Hence, the Be-Te Raman doublet consists of two harmonic (bond-stretching, electric) 1D-oscillators ($u$_1_,$E$) and ($u$_2_, $E$). Both oscillators are characterized by the same reduced mass $\mu$ and the same effective charge $Z$, on account that they ultimately refer to the same bond-stretching. Now, different spring-like restoring force constants (denoted $k$_1_ and $k$_2_, correspondingly) are used depending on the local environment. Different damping terms ($\alpha$_1_ and $\alpha$_2_) behind the friction forces are also considered, a priori. The mechanical coupling between oscillators, modeled via an additional spring-like restoring force ($k$’), completes the picture. A schematic view of the electrically ($E$) and mechanically ($k$’) coupled system of 1D-oscillators is given and commented in Ref. 10.

In a classical description of the Raman scattering process, the electric field $E_{las.}$ carried by the incident visible laser beam forces the oscillation of the valence electrons that vibrate naturally in the same (visible) spectral domain. At the zero order of the light-matter interaction, the as-stimulated oscillating electronic dipole scatters light elastically, corresponding to the Rayleigh scattering. At the first-order of the light-matter interaction, corresponding to the Raman scattering, the electronic susceptibility ($\chi$) – that measures the “capacity” of the electrons to follow $E_{las.}$ – is modulated by the passing of an optic phonon, and the light is inelastically scattered at a frequency that deviates from that of the laser by an amount corresponding to the frequency of the involved phonon. In the used Stokes process, the inelastic scattering refers to an energy loss with respect to the laser energy. According to the principle of the Hertz dipole, the Raman cross section scales as the average over a time period (noted $\left\langle\ldots\right\rangle_{t}$) of the fluctuations in time of the (phonon-modulated) electronic dipolar moment squared. The sensitive issue, then, is to express the dipolar moment in question.

For doing so, Hon and Faust^40^ rely on the fact that Raman scattering is essentially a non-linear process, in that the interaction between the visible laser beam ($E_{las.}$) and the revealed elementary excitation, *i.e*., an optic phonon ($u$,$E$) – vibrating in the far-infrared spectral range – is indirect, *i.e*., mediated by the valence electrons, characterized by their displacement ($y$) around their equilibrium position – that interact resonantly with $E_{las.}$. Accordingly, the cited authors consider for the non-linear dipolar moment $P_{NL}$, the most general bilinear (reflecting the non-linearity) form of the dipolar moment obtained by combining on equal footing the phonon and electron ($u$,$y$) displacements defined in the far-infrared (phonon – absence of subscript) and visible (electron – with subscript $v$) spectral ranges, *i.e*., $P_{NL}\sim f(u\cdot y_{v},{u\cdot u}_{v}, y\cdot y_{v},y\cdot u_{v}).$ Phonons are frozen in the visible spectral range (Born-Oppenheimer adiabatic approximation), meaning that $u_{v}=0.$ Out of the remaining terms, $y$ and $y_{v}$ are proportional to the existing macroscopic electric fields in the corresponding spectral ranges, *i.e.*, $E$ and $E_{las.}$, respectively. A simple (linear) combination leads to $P_{NL}\sim\left( a\cdot u+b\cdot E \right)\cdot E_{las.}$. In this expression *a* and *b* refer to $\chi$-modulations via the lattice displacement (${\partial\chi}/{\partial u}$) and the accompanying electric field (${\partial\chi}/{\partial E}$) of an optic mode, referred to as the deformation potential and electro-optic scattering mechanisms, respectively. Out of resonance conditions (between the laser beam and the valence electrons) – as in the current Zn_1-x_Be_x_Te Raman study, $a$ and $b$ can be considered as constants, in a first approximation.

Deporting $E_{las.}$ (related to the laser beam) into the proportionality sign ($\sim$), and generalizing the above expression for $P_{NL}$ valid for one ($u$,$E$) oscillator to the two sub-oscillators forming the Be-Te percolation doublet ($u$_1_,$E$) and ($u$_2_, $E$), leads to the following form of Raman cross section ($RCS$),

$RCS \sim\left\langle P_{NL}^{2} \right\rangle_{t} \sim\left\langle\left( a_{1}\cdot u_{1}+a_{2}\cdot u_{2}+b\cdot E \right)^{2} \right\rangle_{t}$ (1)

By factorizing $b$ and setting $E$=0, we obtain the generic form of the $RCS$ for the purely-mechanical TO modes ($E$=0),

$RCS \sim\left[ \frac{a_{1}}{b}(x_{1}) \right]^{2}{\cdot\left\langle u_{1}^{2} \right\rangle}_{t}+{\left[ \frac{a_{2}}{b}(x_{2}) \right]^{2}\cdot\left\langle u_{2}^{2} \right\rangle}_{t}+\frac{a_{1}}{b}(x_{1})\cdot\frac{a_{2}}{b}(x_{2})\cdot\left\{ \left\langle u_{1}\cdot u_{2} \right\rangle_{t}+\left\langle u_{2}\cdot u_{1} \right\rangle_{t} \right\}$, (2)

where $x_{i}$ represents the fraction of BeTe-like oscillator-$i$ of Zn_1-x_Be_x_Te at composition $x$ (given by the terms in square brackets in Fig. 1). The $\frac{a_{i}}{b}(x_{i})$ ($i$=1,2) terms come into the $i$-related Faust-Henry coefficients^14^ measuring the relative efficiency of the “deformation potential” and “electro-optic” scattering mechanisms. These scale linearly with the parent (BeTe in this case) value^15^ ($C_{F-H}$) along,

$C_{F-H,i}\left( x_{i} \right)=x_{i}\cdot C_{F-H}=\frac{a_{i}}{b}(x_{i})\cdot\frac{Z}{\mu\omega_{i}^{2}(x_{i})}$, (3)

where $\omega_{i}^{2}(x_{i})$ refers to the square TO frequency of the $i$-related TO mode at composition $x$.

The terms averaged in time are estimated by applying the fluctuation-dissipation (Nyquist) theorem^40^ to the following set of equations,

$\left\{ \begin{aligned} \mu\ddot{u}_{1}=-k_{1}u_{1}-\alpha_{1}\dot{u}_{1}-k^{'}\left( u_{1}-u_{2} \right)+ZE+\frac{F}{Nx_{1}} \\ \mu\ddot{u}_{2}=-k_{2}u_{2}-\alpha_{2}\dot{u}_{2}-k^{'}\left( u_{2}-u_{1} \right)+ZE+\frac{G}{Nx_{2}} \\ \varepsilon_{0}\varepsilon_{r}E=\varepsilon_{\infty}E+x_{1}NZu_{1}+x_{2}NZu_{2}+P_{free} \end{aligned} \right.$ (4)

characterizing both the dynamics of each (Be-Te) 1D sub-oscillator, together with the nature of the involved optical mode via the polarization equation, through $\varepsilon_{r}={q^{2}c^{2}}/{\omega^{2}}$ for a TO mode and $\varepsilon_{r}=0$ for a LO mode^S13^. The used parameters were introduced above, except the added vector of so-called generalized external forces $\left( F,G,P_{free} \right)$, defined per unit volume of the crystal (refer to the polarization equation). Hence a renormalization via the number $N$ of chemical bonds (Be-Te in this case) per crystal volume unit co-weighted by the $x_{i}$ oscillator fraction is needed prior incorporation of $\left( F,G \right)$ in the first two equations, written on a per oscillator basis.

A convenient matrix-like description of the above system eventually gives

$\left( \begin{matrix} P_{free} \\ F \\ G \end{matrix} \right)=\left( \begin{matrix} \varepsilon_{0}{(\varepsilon}_{r}-\varepsilon_{\infty}) & -x_{1}NZ & x_{2}NZ \\ -x_{1}NZ & x_{1}N\mu L_{1}(\omega) & -x_{1}N\mu{\omega'}^{2} \\ x_{2}NZ & -x_{2}N\mu{\omega'}^{2} & x_{2}N\mu L_{2}(\omega) \end{matrix} \right)\left( \begin{matrix} E \\ u_{1} \\ u_{2} \end{matrix} \right)$, (5)

considering solutions periodic in time ($t$) for the lattice displacements, *i.e*., in the form $u_{i}(t)\sim e^{j\omega t}$ (the space phase term is omitted on account that Raman scattering operates at $q\sim0$). In this expression, $\omega'$ defined as $\sqrt{{k'}/\mu}$ represents the mechanical coupling between oscillators $i$=(1,2), and $L_{i}\left( \omega\right)=\omega_{i}^{2}(x_{i})+{\omega'}^{2}+j\gamma_{i}\omega-\omega^{2}$, where $\omega_{i}(x_{i})=\sqrt{{k_{i}(x_{i})}/\mu}$ is the eigenfrequency of the bare-uncoupled oscillator $i$ with fraction $x_{i}$ (such dependence for the $k_{i}$-terms is omitted above, for clarity) and $\gamma_{i}={\alpha_{i}}/\mu$ stands for the individual phonon damping.

According to the fluctuation-dissipation theorem^40^, $\left\langle u_{i}^{2} \right\rangle_{t}\sim Im(T_{ii})$ and $\left\langle u_{i}\cdot u_{j} \right\rangle_{t}\sim Im(T_{ij})$, where $T_{ij}$ is the ($i$,$j$)-element of the linear response matrix $\tilde{T}$ between the stimulus vector $S=\left( P_{free},F,G \right)$ applied (virtually) to the crystal and the response vector $R=\left( E, u_{1},u_{2} \right)$ of the crystal, *i.e*., $\tilde{T}$ $\cdot S=R$. Incidentally, the imaginary character in the above expressions is what motivated the incorporation of a friction force into the force assessment per oscillator.

The matrix in Eq. (5) referring to $\tilde{T}^{-1}$ has to be inverted before use. This leads to a preliminary form of the $RCS$ for the mechanically-coupled TO-like Be-Te doublet of Zn_1-x_Be_x_Te, given by

$RCS\sim\frac{1}{\varepsilon_{\infty}\Omega^{2}}\cdot Im\left\{ \frac{\left( \varepsilon_{r}-\varepsilon_{\infty} \right)\cdot\left( x_{2}\omega_{2}^{4}L_{1}\left( \omega\right)+x_{1}\omega_{1}^{4}L_{2}\left( \omega\right)+\omega_{1}^{2}\omega_{2}^{2}{\omega^{'}}^{2} \right)-x_{1}x_{2}\varepsilon_{\infty}\Omega^{2}\left( \omega_{1}^{2}-\omega_{2}^{2} \right)^{2}}{\left( \varepsilon_{r}-\varepsilon_{\infty} \right)\cdot\left( L_{1}\left( \omega\right)L_{2}\left( \omega\right)-{\omega^{'}}^{4} \right)-\varepsilon_{\infty}\Omega^{2}\left( x_{1}L_{1}\left( \omega\right)+x_{2}L_{2}\left( \omega\right)+{\omega^{'}}^{2} \right)} \right\}$, (6)

where $\Omega^{2}=\frac{NZ^{2}}{\varepsilon_{\infty}\varepsilon_{0}\mu}$ identifies with $\left( \omega_{L}^{2}-\omega_{T}^{2} \right)$ of BeTe. The $x_{i}$-dependence of the $\left\{ \omega_{i},L_{i} \right\}$ parameters is omitted for clarity.

Considering further that $\varepsilon_{r}\to\infty$ for a purely-mechanical TO mode probed in the backscattering geometry (on the basis of the finite/large transferred $q$ value), the above $RCS$ simplifies to

$RCS\sim\frac{1}{S_{BeTe}\omega_{T}^{2}}\cdot Im\left\{ \frac{x_{2}\omega_{2}^{4}L_{1}\left( \omega\right)+x_{1}\omega_{1}^{4}L_{2}\left( \omega\right)+\omega_{1}^{2}\omega_{2}^{2}{\omega^{'}}^{2}}{L_{1}{\left( \omega\right)L}_{2}\left( \omega\right)-{\omega^{'}}^{4}} \right\}$. (7)

This formula is directly exploited in Fig. 1c, where the analysis is arbitrarily placed at the Zn-Te bond percolation threshold (x=0.81). $S_{BeTe}$ is the oscillator strength of the (TO,LO) optic mode of BeTe, defined as^S16^ $S_{BeTe}=\frac{\varepsilon_{\infty}\Omega^{2}}{\omega_{T}^{2}}$. $\omega'$ is taken equal to 50 cm^-1^ (Sec. I.3.a.), the $\omega_{i}$-terms are pressure-dependent, and the same minimal $\gamma_{i}$-damping (1 cm^-1^) is used for both oscillators for optimal resolution of neighboring coupled features on approach to the resonance. The pressure-dependence of the pre-factor is determined considering the individual variations of the constituting $\omega_{T}$ and $\omega_{L}$ frequencies^S17^ and $\varepsilon_{\infty}$ parameter^S18^ of BeTe, available in the literature. An overview of the pressure-induced dependence of the $RCS$ depending on the $\omega'$ value is reported in Fig. S10a.

For the sake of completeness, we further provide in Fig. S10b the pressure-dependence of the $RCS$ related to the Zn-Te percolation doublet of Zn_1-x_Be_x_Te, symmetrically calculated at the Be-Te bond percolation threshold (x=0.19). In this case the lower and upper modes are minor and dominant at ambient pressure (conversely to the Be-Te situation at x=0.81). By analogy with the Be-Te doublet, the resonance of the bare-uncoupled Zn-Te TO submodes is considered to occur at 5 GPa, and $\omega'$ is arbitrarily taken at 20 cm^-1^, representing roughly 10% of the TO frequency of ZnTe. The pressure dependence of ($\omega_{T}$, $\omega_{L}$)^S19^ and of $\varepsilon_{\infty}$^S19,S20^ for ZnTe, are taken from the literature.

Last, the $RCS$ due to the overdamped/mechanically-decoupled Be-Te oscillators at the same composition (x=0.19), depicted in Fig. 1d, is achieved by taking $\omega^{'}=0$ in Eq. (7), resulting in the traditional $RCS$ for two independent TO oscillators,

$RCS\sim\frac{1}{S_{BeTe}\omega_{T}^{2}}\cdot Im\left\{ \frac{x_{1}\omega_{1}^{4}}{L_{1}\left( \omega\right)}+\frac{x_{2}\omega_{2}^{4}}{L_{2}\left( \omega\right)} \right\}\sim Im\left\{ \varepsilon_{r}(\omega,x) \right\}$. (8)

A linear dead loss of oscillator strength under pressure is considered for the lower Be-Te oscillator until its total extinction at the resonance, in reference to the achievement of a phonon exceptional point at this limit (see main text).

**Supplementary Information – only References**

S1. Dicko, H. *et al*. Near-forward/high-pressure-backward Raman study of Zn_1-x_Be_x_Se (x~0.5) – evidence for percolation behavior of the long (Zn-Se) bond. *J. Raman. Spectrosc.* **47**, 357–367 (2015).

S2. Pradhan, G.K. *et al*. The phonon percolation scheme for alloys: Extension to the entire lattice dynamics and pressure dependence. *Japan. J. Appl. Phys.* **50**, 05FE02/1–4 (2011).

S3. Pradhan, G.K. *et al*. Pressure induced metallization in Zn_1-x_Be_x_Se ternary mixed crystals. *J. Phys.: Conf. Series* **377**, 012019/1–5 (2012).

S4. Dewaele, A., Datchi, F., Loubeyre, P. & Mezouar, M. High pressure – high temperature equations of state on neon and diamond. *Phys. Rev. B* **77**, 094106/1–9 (2008).

S5. Hammersley A. P., Svensson, S. O., Hanfland, M., Fitch, A. N. & Hausermann, D. Two-dimensional detector software: from real detector to idealized image or two-theta scan. *High Press. Res.* **14**, 235–248 (1996).

S6. Pellicer-Porres, J. *et al*. High-pressure phase diagram of ZnSe_x_Te_1-x_ alloys. *Phys. Rev. B* **71**, 035210 (2005).

S7. San-Miguel, A., Polian, A., Gauthier, M. & Itié, J.P. ZnTe at high pressure: X-ray-absorption spectroscopy and x-ray diffraction studies. *Phys. Rev. B* **48**, 8683–8693 (1993).

S8. Luo, H. *et al*. Phase transformation of BeSe and BeTe to the NiAs structure at high pressure. *Phys. Rev. B* **52**, 7058–7064 (1995).

S9. Zamir, D., Beshah, K., Becla, P., Wolff, P. A. & Griffin, R. G. Nuclear magnetic resonance studies of II-VI semiconductor alloys. *J. Vac. Sci. Technol. A* **6**, 2612–2613 (1988).

S10. Edwards, T.G., Gjersing, E.L., Sen, S., Currie, S.C. & Aitken, B.G. ^125^Te NMR chemical shifts and tellurium coordination environments in crystals and glasses in the Ge-As-Sb-Te system. *J. Non-Cryst. Solids* **357**, 3036–3041 (2011).

S11. García, A. *et al*. Siesta: Recent developments and applications. *J. Chem. Phys.* **152**, 204108/1–31 (2020).

S12. Moreno, J. & Soler, J. Optimal meshes for integrals in real- and reciprocal-space unit cells. *Phys. Rev. B* **45**, 13891–13898 (1992).

S13. Dicko, H. *et al*. Defect-induced ultimately fast volume phonon-polaritons in the wurtzite Zn_0.74_Mg_0.26_Se mixed crystal. *Sci. Rep.* **9**, 7817/1–8 (2019).

S14. Cardona, M. in *Light scattering in Solids II, Topics in Applied Physics, Vol. 50*, (eds by Cardona M. & Güntherodt, G., Springer-Verlag, Berlin, 1982), Chap. 2 – Resonance phenomena, p. 60.

S15. Groenen, J., Carles, R., Landa, G. Guerret-Piécourt, C., Fontaine, C. & Gendry, M. Optical-phonon behavior in Ga_1-x_In_x_As: The role of microscopic strains and ionic plasmon coupling. *Phys. Rev. B* **58**, 10452–10462 (1998).

S16. Chang, I.F. & Mitra, S.S. Application of a Modified Random-Element-Isodisplacement Model to Long-Wavelength Optic Phonons of Mixed Crystals. *Phys. Rev.* **172**, 924–933 (1968).

S17. Mameri Z., Zaoui, A., Belabbes, A. & Ferhat M. Pressure effects on the phonon modes in beryllium chalcogenides. *Mater. Chem. Phys.* **123**, 343–346 (2010).

S18. Khenata, R., Bouhemadou, A., Hichour, M., Baltache, H., Rached, D., Rérat, M. Elastic and optical properties of BeS, BeSe and BeTe under pressure. M. Solid-State Electron. **50**, 1382–1388 (2006).

S19. Khenata, R., Bouhemadou, A., Sahnoun, M., Reshak, A.H., Baltache, H. and Rabah, M. Elastic, electronic and optical properties of ZnS, ZnSe and ZnTe under pressure. *Comput. Mat. Sci.* **38**, 29–38 (2006).

S20. Nourbakhsh, Z. Structural, electronic and optical properties of ZnX and CdX compounds (X=Se, Te and S) under hydrostatic pressure. *J. Alloys Compounds* **505**, 698–711 (2010).


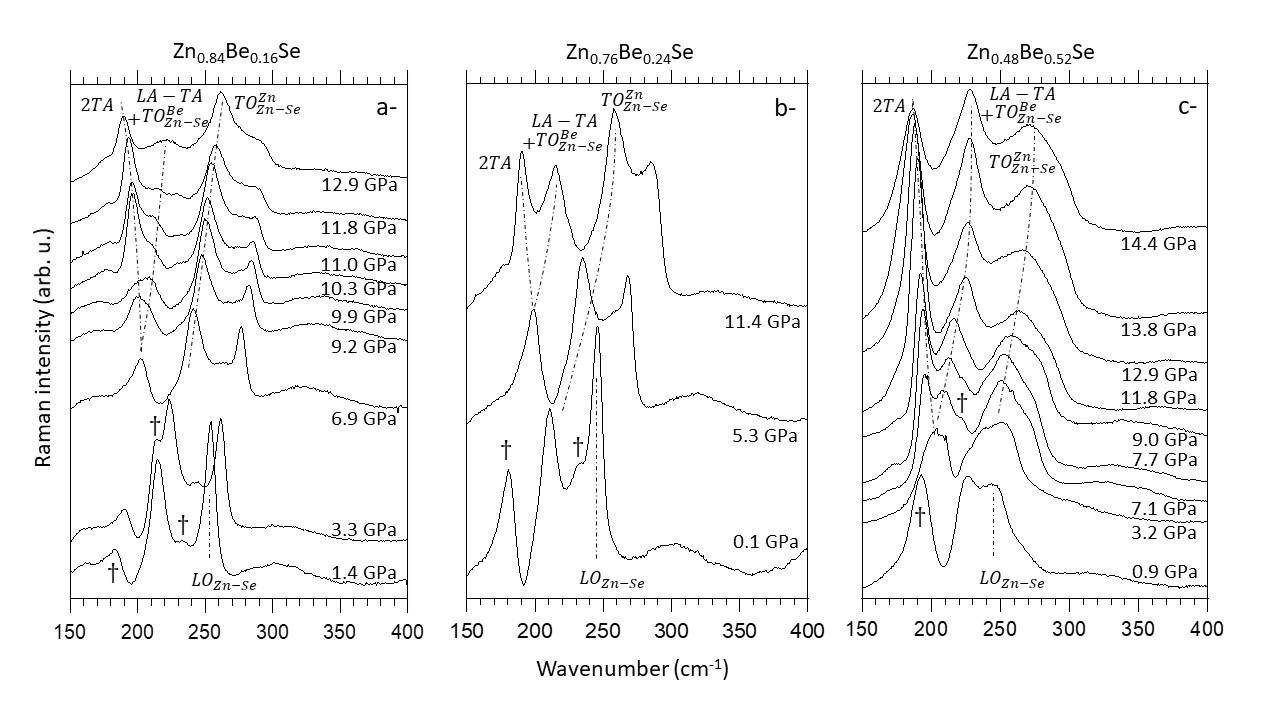


**Figure S1 ⏐ High-pressure Raman spectra of bulk Zn_1-x_Be_x_Se crystals.** Data collected in the native zincblende structure in the backscattering geometry using the 532.0 nm laser line. **a)** x=0.16. **b)** x=0.24. **c)** x=0.52. Additional Zn_0.76_Be_0.24_Se Raman spectra depending on pressure completing the current series are given in Ref. 17. The dotted curves are guidelines for the eyes indicating an apparent “divergence” of the Zn-Te TO doublet under pressure, at any composition. The crosses mark complex Raman features at least partially involved with zone-edge two-phonon acoustic bands at small/moderate pressure.


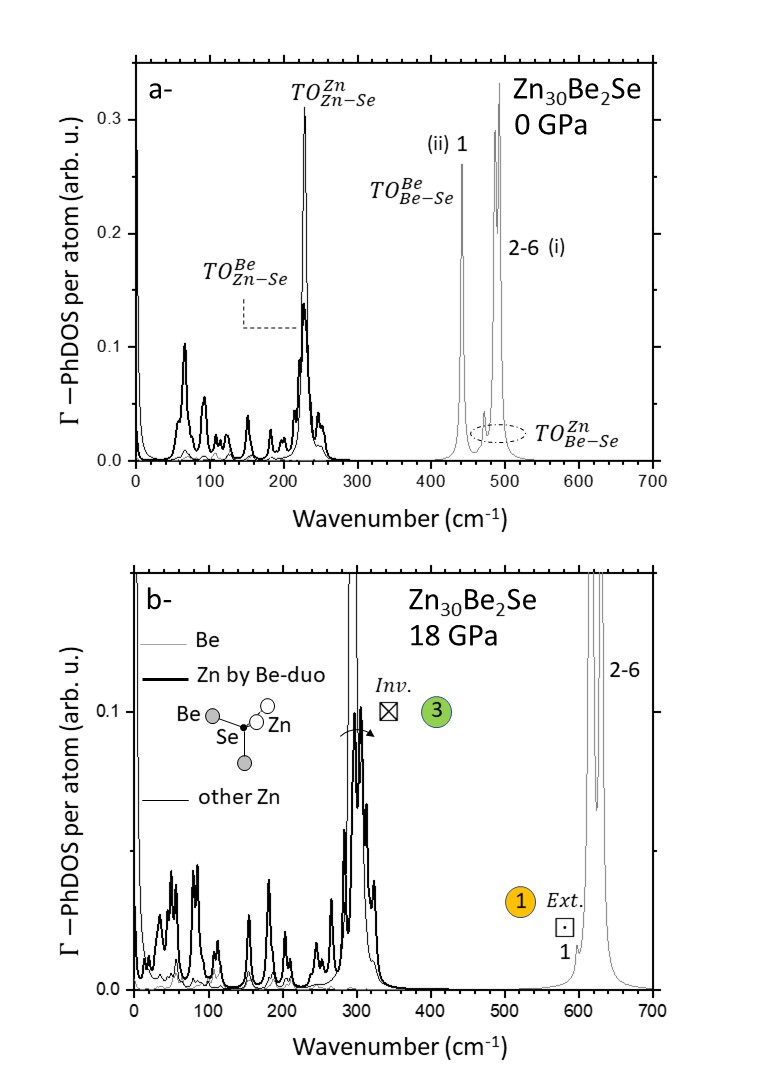


**Figure S2 ⏐ *Ab initio* (SIESTA code) high-pressure Zn_1-x_Be_x_Se phonon spectra (x~0).** $\Gamma$-projected PhDOS normalized per atom of a prototypical Zn_30_Be_2_Se_32_ zincblende-type supercell containing a Be-duo, the prototypical percolation-type impurity motif. **a)** Ambient pressure (0 GPa). **b)** 18 GPa. In the Be-related spectral range, the individual vibration modes of the Be-duo are labeled according to the used terminology in Fig. 3 (*e.g*., ii covering the 2-6 modes), for a direct comparison. In the Zn-spectral range, a distinction is made between the two Zn atoms connected to the Se atom bridging the Be-duo, and the remaining ones.


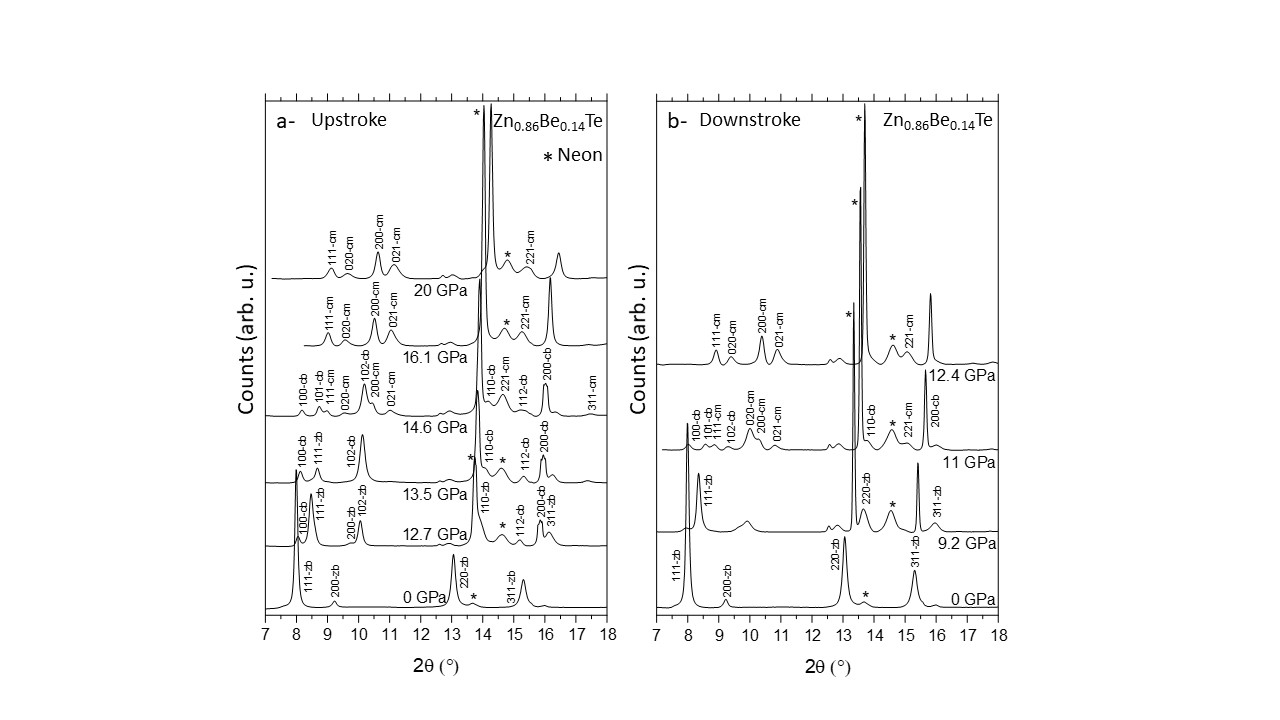


**Figure S3 ⏐ Zn_086_Be_0.14_Te high-pressure x-ray diffraction spectra.** Selection of X-ray diffractograms of Zn_086_Be_0.14_Te depending on pressure taken at the CRISTAL beamline of synchrotron SOLEIL using the 0.485 $Å$ x-ray radiation. **a)** Upstroke regime (pressure increase). **b)** Downstroke regime (pressure decrease). Stars mark diffraction lines due to Neon used as the pressure transmitting medium. The individual diffractions peaks are labeled via the relevant miller indices of the successive zincblende (Zb), Cinnabar (Cb) and Cmcm (abbreviated Cm) phases, as specified.


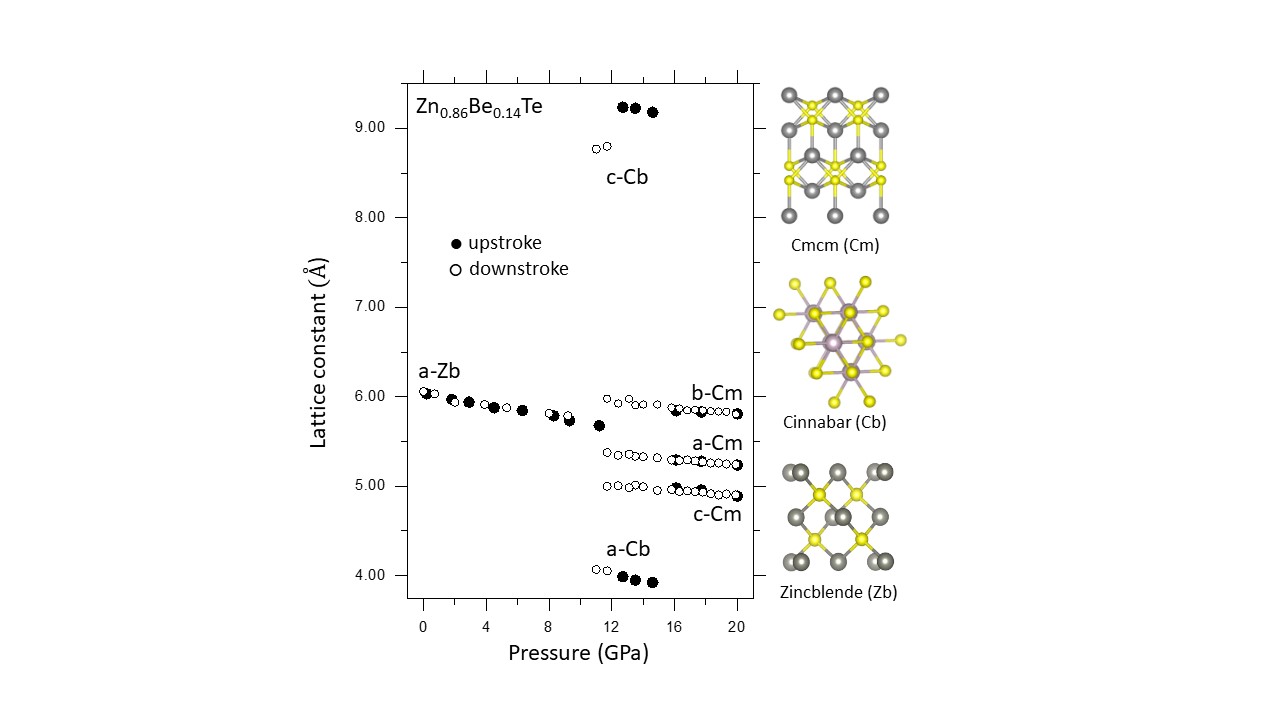


**Figure S4 ⏐ Pressure dependence of the Zn_086_Be_0.14_Te lattice constant(s).** X-ray diffraction measurements of the Zn_086_Be_0.14_Te lattice constant(s) depending on pressure in the successive native-zincblende (Zb), transient-Cinnabar (Cb) and high-pressure Cmcm (abbreviated Cm) crystal phases, as sketched out, for clarity. A distinction is made between data taken in the upstroke (filled symbols) and downstroke (hollow symbols) regimes.


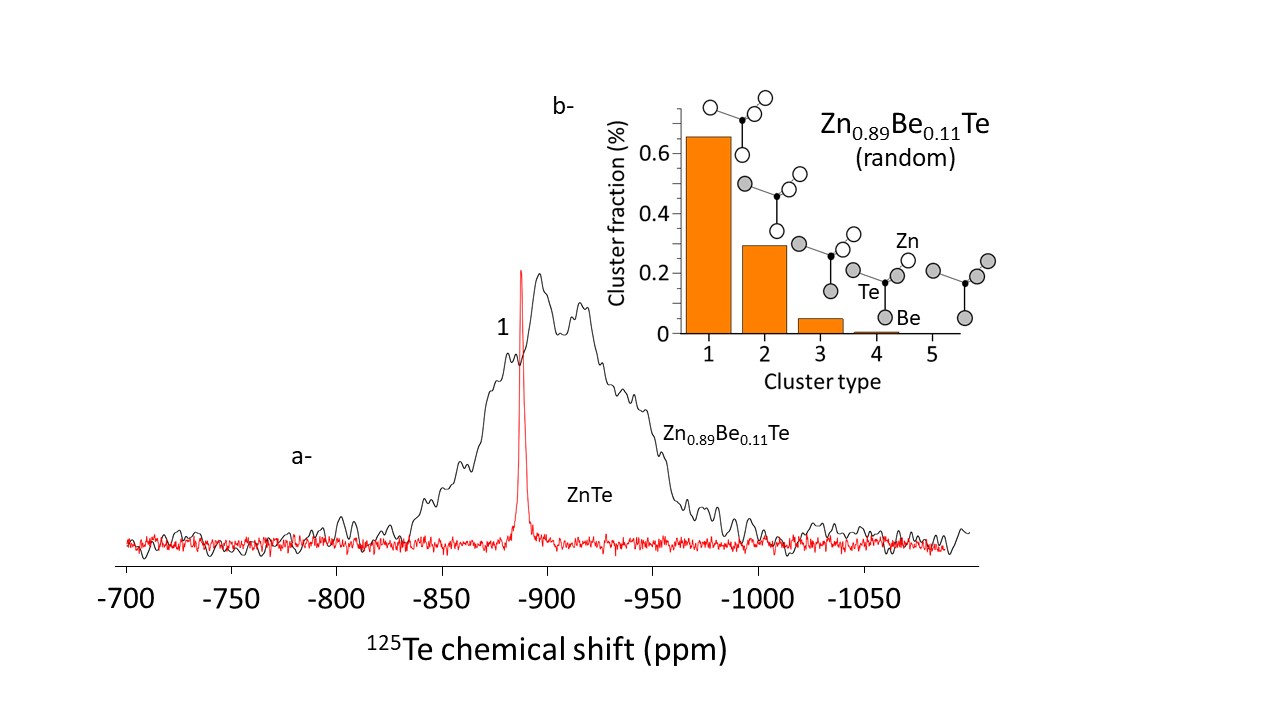


**Figure S5 ⏐ ^125^Te NMR signal of Zn_0.89_Be_0.11_Te.** **a)** One-dimensional ^125^Te NMR spectrum of Zn_0.89_Be_0.11_Te in direct acquisition with ~5T_1_ recycle delay taken over 60h. The corresponding ^125^Te NMR ZnTe signal is added for reference purpose. **b)** Binomial distribution of the five possible Te-centered tetrahedron units (sketched out) in case of a random Zn$\leftrightarrow$Be substitution.


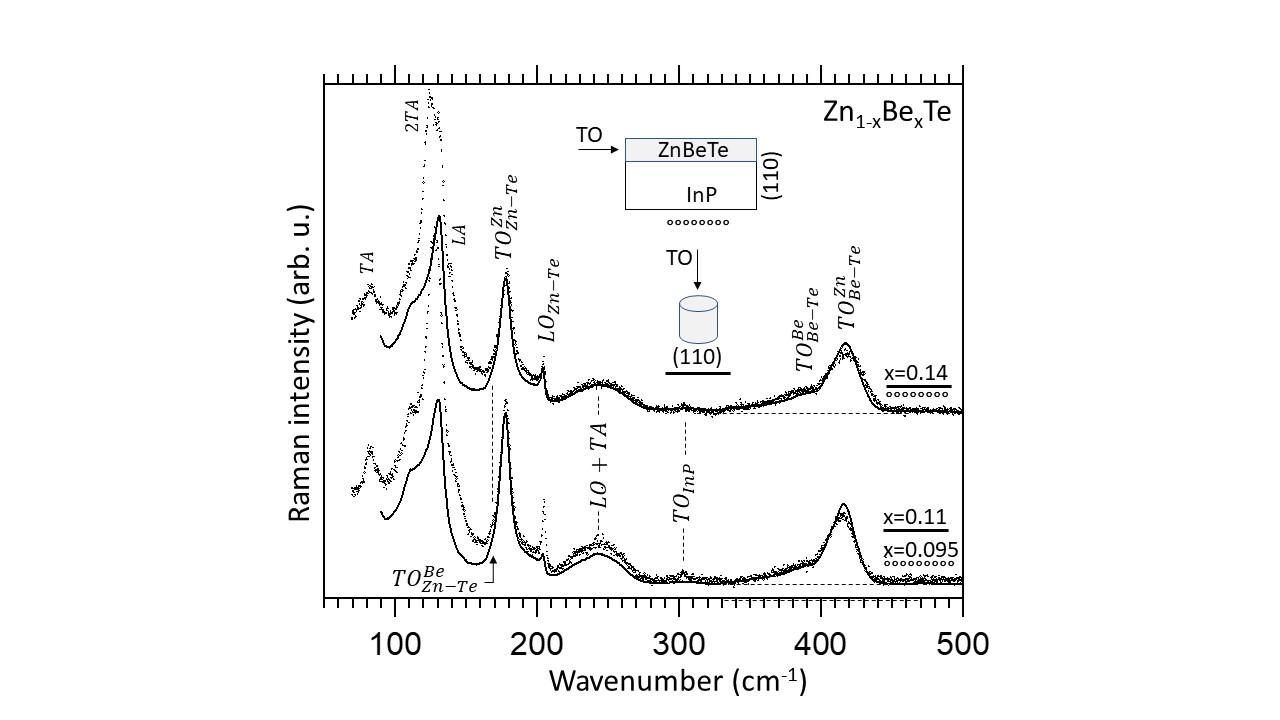


**Figure S6 ⏐ TO-like Zn_1-x_Be_x_Te Raman spectra – epilayers vs. bulk crystals.** Comparison between pure-TO Raman spectra taken on Zn_1-x_Be_x_Te epilayers (symbols, data taken from Ref. 7, "© IOP Publishing. Reproduced with permission. All rights reserved") and our current bulk crystals (curves) of (nearly) the same composition by using the 647.1 nm and 632.8 nm laser lines in the backscattering geometry operated at normal incidence/detection on (110)-edge and ‑cleaved crystal faces, respectively.


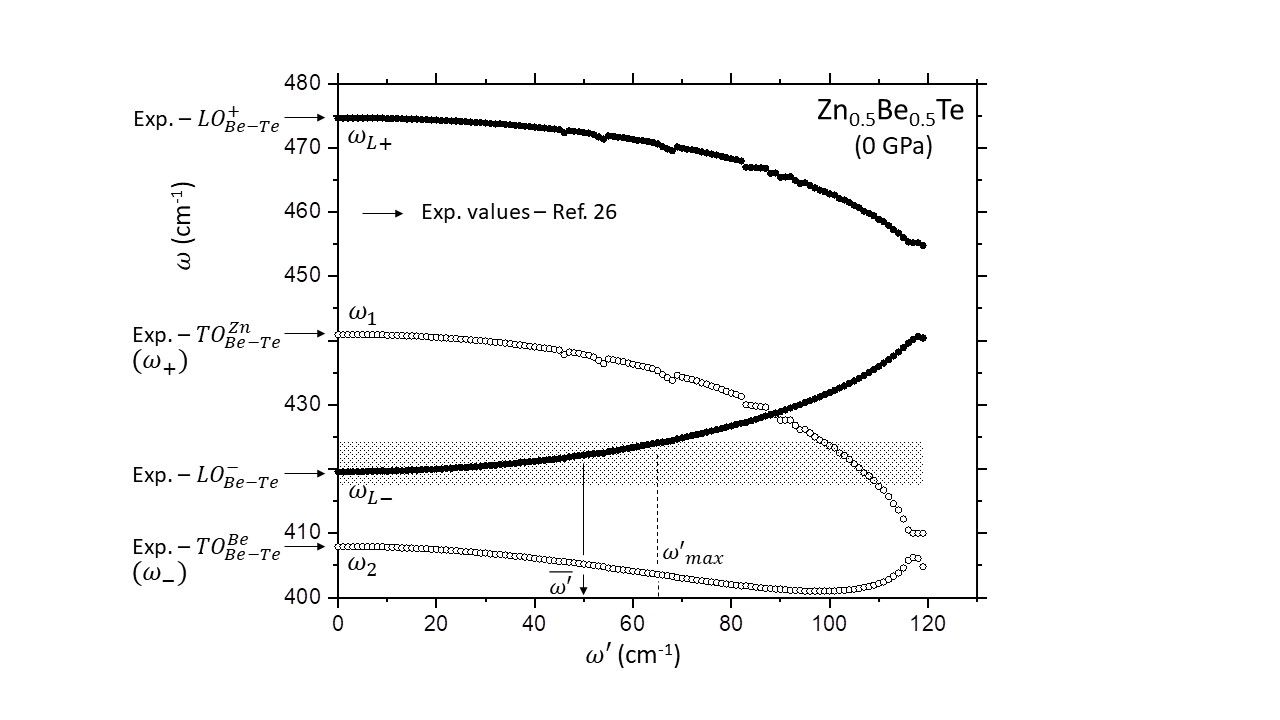


**Figure S7 ⏐ Raman frequencies of Zn_0.5_Be_0.5_Te depending on mechanical coupling.** Predicted bare-uncoupled frequencies ($\omega_{1},\omega_{2}$) of the purely-mechanical TO modes of Zn_0.5_Be_0.5_Te behind the experimental coupled TO frequencies ($\omega_{-},\omega_{+}$) in their dependence on mechanical coupling ($\omega'$), and corresponding variations of the coupled LO frequencies ($\omega_{L,-},\omega_{L+}$). The experimental $\omega_{L,-}$ frequency (taken from Ref. 26, together with alternative experimental values, pointed by arrows), though marred by a large error (shaded area) is used as a marker to estimate $\omega'$.


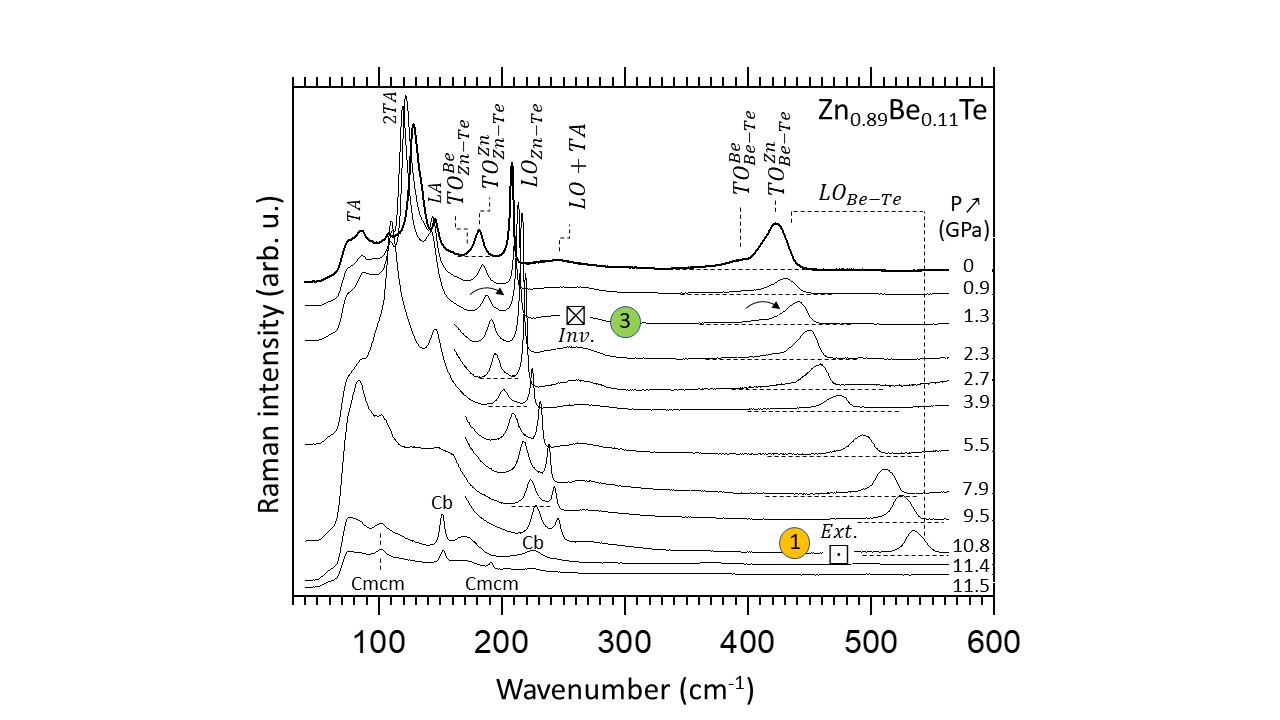


**Figure S8 ⏐ Zn_0.89_Be_0.11_Te high-pressure Raman spectra.** Selection of high-pressure Zn_0.89_Be_0.11_Te Raman spectra taken with the 632.8 nm laser line in the upstroke regime (up-pointing arrow). The spectrum taken at ambient pressure (thick curve) offers a convenient reference. The pressure-induced inversion ($⊠$) and extinction ($⊡$) processes in the Zn-Te and Be-Te ranges are emphasized (curved arrows).


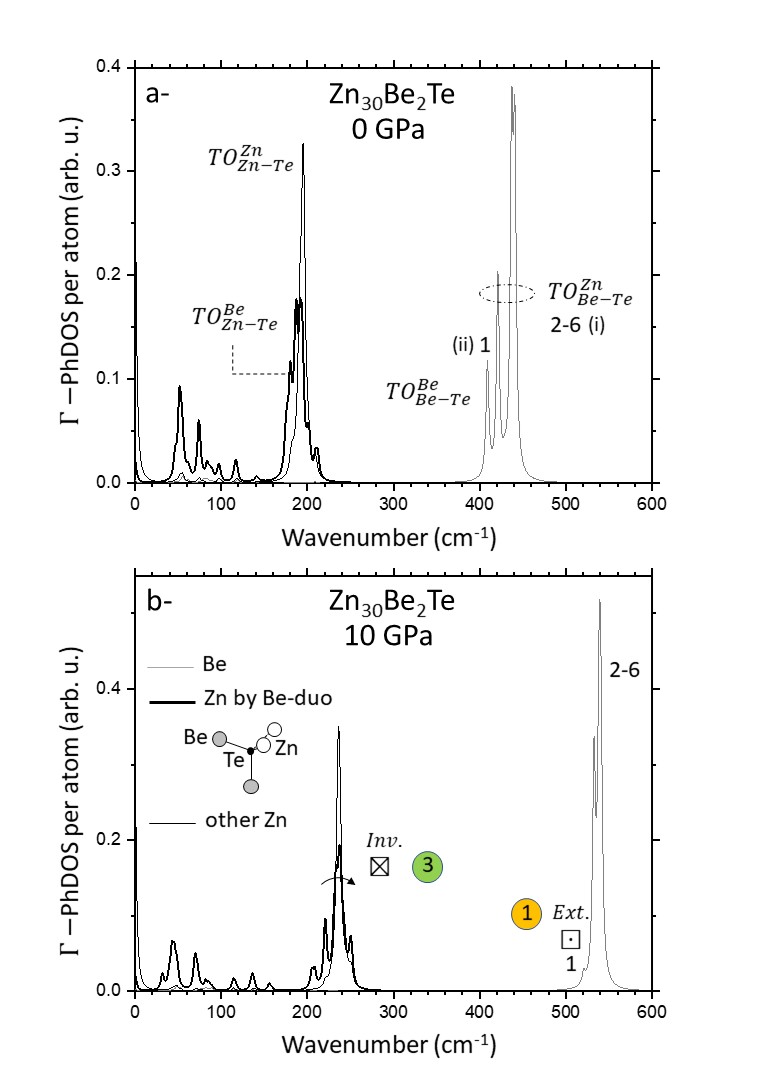


**Figure S9 ⏐ *Ab initio* (SIESTA code) high-pressure Zn_1-x_Be_x_Se phonon spectra (x~0).** $\Gamma$-projected PhDOS normalized per atom of a prototypical Zn_30_Be_2_Te_32_ zincblende-type supercell containing a Be-duo, the prototypical percolation-type impurity motif. **a)** Ambient pressure (0 GPa). **b)** 10 GPa. In the Be-related spectral range, the individual vibration modes of the Be-duo are labeled according to the used terminology in Fig. 3 (*e.g.*, i standing for mode 1), for a direct comparison. In the Zn-spectral range, a distinction is made between the two Zn atoms connected to the Te atom bridging the Be-duo, and the remaining ones.


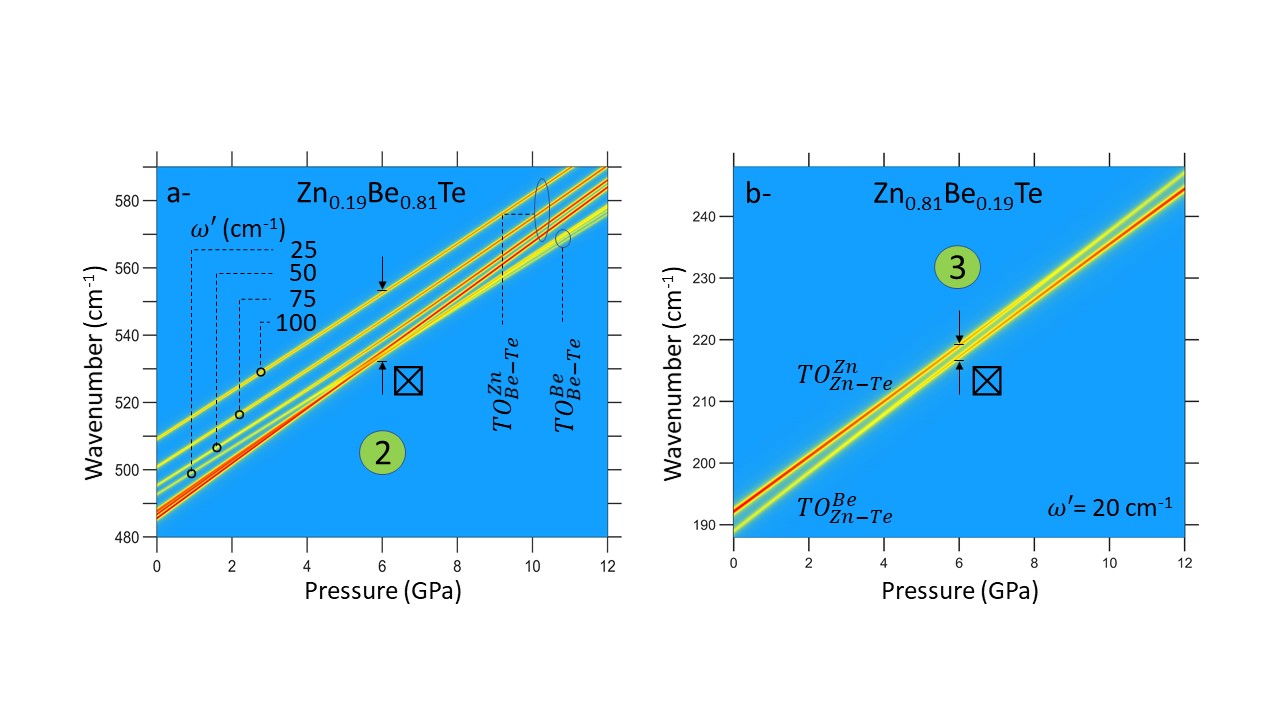


**Figure S10 ⏐ Free-coupling regimes in Zn-Te and Be-Te spectral ranges.** **a)** Theoretical insight into the pressure-induced free-coupling taking place between the two Be-Te TO-submodes of Zn_1-x_Be_x_Te at the Zn-Te bond percolation threshold (x=0.81) depending on the amount of mechanical coupling $\omega'$ varying between 25 cm^-1^ and 100 cm^-1^, as indicated. On both sides of the resonance, the lower Be-Te feature is upward shifted by increasing $\omega'$ (not shown) as the upper one (shonw). **b)** Corresponding insight into the Zn-Te doublet at the Zn-Te bond percolation threshold (x=0.19) using $\omega'$=20 cm^-1^. The resonance of the bare-uncoupled oscillators is considered to occur at 5 GPa in both cases. In both panels red/yellow glow reflect large/small Raman intensities.
